# Impact of early dexmedetomidine administration on short‐ and long‐term outcomes in critically traumatic brain injury patients: A retrospective study using the MIMIC‐IV database

**DOI:** 10.1002/ibra.70015

**Published:** 2026-03-11

**Authors:** Yang Long, Long‐Zhen Liu, Xiao‐Xue Tan, Yong‐Hu Zhang, Cheng‐Xi Liu, Shan Liu, Wen‐Jie Liu

**Affiliations:** ^1^ Department of Anesthesiology, The Second Affiliated Hospital, Hengyang Medical School University of South China Hengyang China; ^2^ Department of Emergency, The Second Affiliated Hospital, Hengyang Medical School University of South China Hengyang China; ^3^ The Second Affiliated Hospital, National Regional Key Cities and Sub‐central Cities in Hunan Province Hengyang Academician and Expert International Academic Exchange High Tech University, Building A First‐class Discipline for Trauma and Critical Care, Hengyang Medical School University of South China Hengyang China

**Keywords:** early dexmedetomidine administration, MIMIC‐IV, survival outcomes, traumatic brain injury

## Abstract

Traumatic brain injury (TBI) causes significant mortality. Dexmedetomidine (DEX) shows neuroprotective potential in animals, but clinical evidence remains inconsistent. We evaluated the impact of early DEX, initiated within 48 h of admission with a treatment duration of at least 4 h, on survival in intensive care unit (ICU) patients with TBI using the Medical Information Mart for Intensive Care‐IV (MIMIC‐IV) database. Outcomes included 28‐day, hospital, and 1‐year mortality, analyzed *via* propensity score matching (PSM), multivariable Cox models, and subgroup analyses. Of 2378 patients, 241 received DEX. After PSM (235 pairs), early DEX use significantly reduced 28‐day (HR 0.45, 95% CI 0.30–0.69, *p* < 0.001) and hospital mortality (HR 0.25, 95% CI 0.15–0.42, *p* < 0.001). These results remained robust across sensitivity analyses. Similarly, 1‐year mortality decreased (HR 0.64, 95% CI 0.47–0.87, *p* < 0.01) and further supported by the Boruta algorithm, although inverse probability of treatment weighting analysis showed only a non‐significant trend (*p* = 0.08). Survival benefits were more pronounced in patients aged <65 and those requiring mechanical ventilation. In conclusion, early DEX use is associated with improved short‐ and long‐term survival in ICU patients with TBI, particularly in younger individuals and those requiring mechanical ventilation. Randomized controlled trials are warranted to establish causality.

## Graphical abstract

Our retrospective analysis of the Medical Information Mart for Intensive Care‐IV (MIMIC‐IV) database suggests that early use of dexmedetomidine is associated with lower short‐term (28‐day and in‐hospital) and long‐term (1‐year) mortality in patients with traumatic brain injury. Abbreviation: ICU, intensive care unit; ICU LOS, ICU length of stay; DEX, dexmedetomidine; MIMIC‐IV, Medical Information Mart for Intensive Care‐IV.

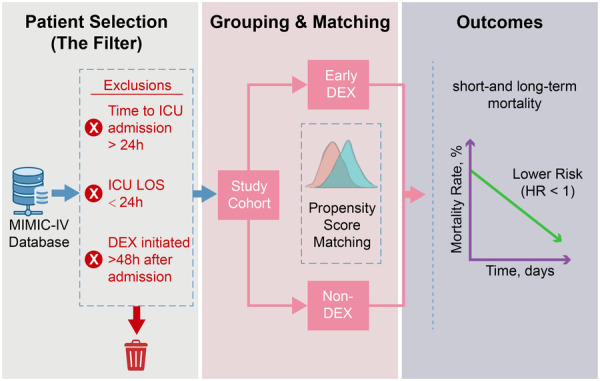

## 1 INTRODUCTION

Traumatic brain injury (TBI) refers to a brain injury resulting from a sudden impact, collision, or other direct trauma to the head, leading to alterations in brain function.^1^ Documented TBI represents a substantial public health burden in the United States, accounting for approximately 2.8 million emergency department visits, 223,000 hospitalizations, and 60,611 deaths each year.^2^ The high prevalence and associated disability of TBI impose a significant burden on public health systems.^3^ TBI is frequently associated with secondary injuries, including cerebral edema, hemorrhage, and ischemia, which often result in a poorer prognosis, underscoring the importance of effective management strategies.^4^ Current treatment approaches for TBI patients in intensive care unit (ICU) focus on stabilizing hemodynamics and systemic oxygenation, reducing intracranial pressure, and managing cerebral vasoconstriction.^5^ However, these interventions remain limited in their efficacy, particularly for patients with moderate to severe TBI.^6^

Dexmedetomidine (DEX) is a potent agonist that selectively targets α2‐adrenergic receptors. It is commonly used in intensive care settings to induce sedation and provide pain relief for critically ill patients.^7^ Recent studies have revealed that DEX not only exerts sedative effects but also demonstrates neuroprotective properties.^8^, ^9^ An animal study shows that DEX can reduce cerebral edema and neurological impairment by modulating anti‐apoptotic cellular mechanisms.^8^ In addition, another animal study showed that DEX reduced neutrophil infiltration, microglia activation, and pro‐inflammatory factor release.^9^ Some researchers have further found that the cerebroprotective effects of DEX may be mediated through activation of α2 adrenergic receptors, which inhibit inflammatory responses in ischemic brain tissue.^10^

DEX has been shown to mitigate secondary brain injury and neuroinflammation in certain animal models of TBI. However, clinical evidence supporting its benefit in TBI patients remains inconsistent. Xu et al. reported improved hospital and 6‐month survival outcomes with DEX use in TBI patients^11^; however, their study included all patients receiving DEX during hospitalization without distinguishing administration timing. This lack of specificity may potentially dilute survival benefits and introduce selection bias in a heterogeneous sample. Conversely, Liu et al. explored early DEX use in critically ill adults with moderate‐to‐severe TBI requiring mechanical ventilation, focusing exclusively on short‐term prognosis.^12^ This narrow scope leaves long‐term outcomes unaddressed, and while DEX is often studied in mechanical ventilation‐dependent patients, its potential benefits in other TBI populations, such as those not requiring ventilatory support, warrant further investigation. These gaps highlight the need to assess the optimal timing and broader applicability of DEX in TBI management. We aim to evaluate the effects of early DEX administration on survival outcomes across multiple time points in ICU patients with TBI and to identify subgroups most likely to benefit.

## 2 METHODS

### 2.1 Data design and data source

This retrospective cohort study utilized the MIMIC‐IV (version 3.1) database, containing de‐identified ICU patient data from Beth Israel Deaconess Medical Center (2008–2022).^13^ The database is approved for research by the Institutional Review Board (IRB) of Beth Israel Deaconess Medical Center and Massachusetts Institute of Technology (MIT); additional IRB approval and patient consent were not required due to de‐identification. Data were extracted and analyzed using structured query language (SQL) via PostgreSQL.

### 2.2 Study population

We included adult patients (≥18 years) with TBI initially admitted to the ICU within 24 h of hospital admission, identified by International Classification of Diseases (ICD) 9 and ICD‐10 codes for TBI, including traumatic intracranial hemorrhage (e.g., subdural hematoma), cerebral laceration, cerebral contusion, and diffuse brain injury. Patients were excluded if their ICU length of stay (ICU‐LOS) was ≤24 h or if they had multiple ICU stays (only the first ICU admission was analyzed). The exposure group consisted of patients who received DEX within 48 h of hospital admission with an infusion duration of ≥4 h. The control group comprised TBI patients who did not receive DEX during their hospital stay.

### 2.3 Data collection

Data were extracted from MIMIC‐IV using SQL queries. Baseline variables included age, sex, race, and comorbidities (heart failure [HF], renal disease, chronic obstructive pulmonary disease, stroke, malignancy), assessed *via* ICD codes. Disease severity was quantified using the acute physiology score III (APS III), sequential organ failure assessment (SOFA) score, and Charlson comorbidity index (CCI). Interventions within the first 24 h of ICU admission (vasopressors, midazolam, propofol, fentanyl) and vital signs (mean arterial pressure [MAP], heart rate [HR], peripheral oxygen saturation [SpO_2_], Glasgow Coma Scale [GCS]) were recorded. Laboratory parameters obtained within the first 24 h of ICU admission included white blood cell count, hemoglobin, platelet count, glucose, electrolytes (sodium, potassium, bicarbonate, chloride), blood urea nitrogen (BUN), lactate, and coagulation profile (international normalized ratio [INR], prothrombin time [PT], activated partial thromboplastin time [APTT]). Details of DEX administration (duration and timing) and clinical events (cerebral surgery, sepsis, ventilator‐associated pneumonia [VAP]) were collected. Cerebral surgery encompassed craniotomy and less invasive procedures, such as lateral ventricle puncture, which do not require skull opening.

### 2.4 Outcomes

The primary outcome was 28‐day mortality. Secondary outcomes included hospital mortality and 1‐year mortality.

### 2.5 Statistical analysis

Continuous variables were reported as means (standard deviations [SD]) or medians (interquartile ranges [IQR]), depending on normality assessed via the Shapiro–Wilk test, and categorical variables were reported as counts (percentages). Baseline differences between the DEX and control groups were compared using *t*‐tests or Mann–Whitney *U* tests for continuous variables and Chi‐square or Fisher's exact tests for categorical variables. Missing data were handled with multiple imputation using the mice package in R. Propensity score matching (PSM) was applied to balance baseline characteristics between groups, using a 1:1 nearest‐neighbor algorithm with a caliper of 0.1; matching adequacy was confirmed by standardized mean differences (SMD) < 0.1, indicating balance. Kaplan–Meier curves and log‐rank tests were used to evaluate survival differences. Cox proportional hazards models estimated the association between DEX use and outcomes, reporting hazard ratios (HR) and 95% confidence interval (CI); all and unbalanced covariates were included in multivariable models. Sensitivity analyses included: (1) full and unbalanced covariate adjustment with the original cohort, (2) selected covariates identified through the Boruta algorithm (parameters: maximum iterations = 10, seed = 100, *p*‐value threshold = 0.0000001), and (3) multivariate Cox model adjusted with all covariates and inverse probability of treatment weighting (IPTW) within the matched cohort. Subgroup analyses explored effect heterogeneity by age (<65 vs. ≧65 years), GCS (<13 vs. ≧13), SOFA (<5 vs. ≧5), mechanical ventilation use, cerebral surgery, and specific injury types (cerebral laceration, subdural hematoma, and diffuse brain injury). Analyses were performed in R (version 4.4.2), with two‐sided *p* < 0.05 considered statistically significant.

## 3 RESULTS

### 3.1 Baseline characteristics

**Figure 1:**
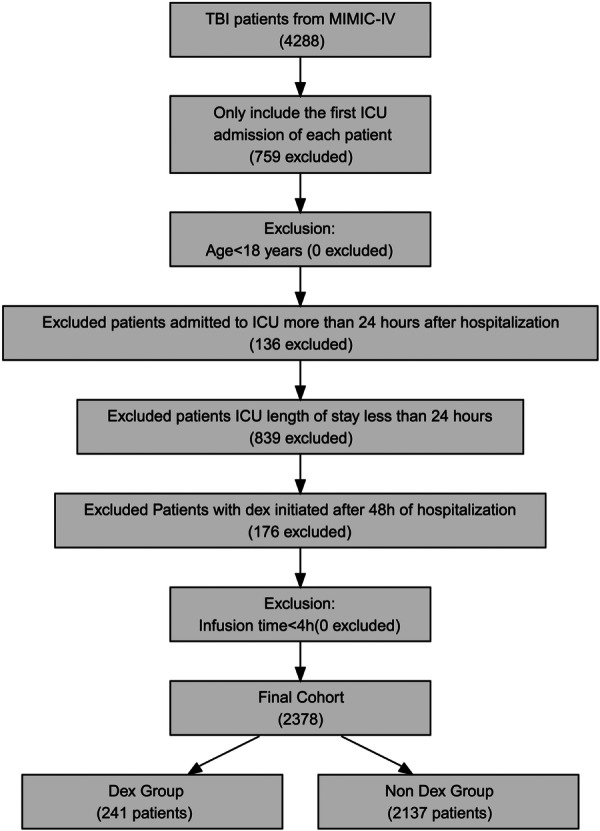
Flowchart of this study. DEX, dexmedetomidine; ICU, intensive care unit; MIMIC‐IV, Medical Information Mart for Intensive Care‐IV; TBI, traumatic brain injury.

The process of this study is detailed in Figure 1, and baseline characteristics of the cohort before and after PSM are presented in Table 1. Initially, the cohort comprised 2378 TBI patients, with 2137 (89.9%) in the non‐DEX group and 241 (10.1%) in the DEX group. Before PSM, the DEX group was notably younger (55.38 vs. 63.18 years, *p* < 0.001), had fewer females (25.31% vs. 37.81%, *p* < 0.001), and a higher proportion of White patients (52.28% vs. 37.39%, *p* < 0.001) (Supplementary Table 1). Additionally, the DEX group had a significantly higher prevalence of diffuse brain injury (8.30% vs. 3.46%, *p* < 0.001). The DEX group exhibited higher SOFA scores (4.06 vs. 3.37, *p* < 0.001) and lower Charlson scores (2.71 vs. 3.40, *p* < 0.001), with no difference in APS III. Within the first 24 h of ICU admission, the DEX group showed increased use of mechanical ventilation (82.57% vs. 41.69%, *p* < 0.001), vasopressors (24.90% vs. 12.73%, *p* < 0.001), and sedatives, including midazolam (13.28% vs. 8.66%, *p* < 0.05), propofol (86.31% vs. 34.44%, *p* < 0.001), and fentanyl (68.88% vs. 28.59%, *p* < 0.001). Vital signs in the first 24 h indicated higher temperature (36.95 vs. 36.76°C, *p* < 0.01) and SpO_2_ (98.64% vs. 97.58%, *p* < 0.001) in the DEX group, with no differences in MAP, HR, or GCS. Laboratory tests within the same period revealed lower platelets (186.18 vs. 200.24 × 10⁹/L, *p* < 0.01), glucose (136.93 vs. 142.21 mg/dL, *p* < 0.01), bicarbonate (22.19 vs. 23.18 mmol/L, *p* < 0.001), BUN (15.60 vs. 17.90 mg/dL, *p* < 0.001), INR (1.15 vs. 1.21, *p* < 0.01), PT (12.43 vs. 13.40 s, *p* < 0.001), and APTT (27.57 vs. 28.95 s, *p* < 0.01) in the DEX group, with no differences in other parameters. Clinical events showed a higher rate of cerebral surgery (96.27% vs. 78.66%, *p* < 0.001), and sepsis (64.32% vs. 40.57%, *p* < 0.001) and VAP (14.94% vs. 5.76%, *p* < 0.001) in the DEX group.

**Table 1:** Baseline characteristics before and after propensity score matching of two cohorts.

	Before matching				After matching			
	Overall (*N* = 2378)	non‐DEX (*N* = 2137)	DEX (*N* = 241)	SMD	Overall (*N* = 470)	non‐DEX (*N* = 235)	DEX (*N* = 235)	SMD
Age, years	**62.39 (21.79)**	**63.18 (21.83)**	**55.38 (20.11)**	**0.372**	54.87 (20.99)	53.86 (21.87)	55.89 (20.07)	0.097
Gender (Female), *n* (%)	**869 (36.54%)**	**808 (37.81%)**	**61 (25.31%)**	**0.271**	115 (24.47%)	54 (22.98%)	61 (25.96%)	0.069
Race (White), *n* (%)	**925 (38.90%)**	**799 (37.39%)**	**126 (52.28%)**	**0.303**	243 (51.70%)	122 (51.91%)	121 (51.49%)	0.009
Cerebral_laceration (YES), *n* (%)	210 (8.83%)	187 (8.75%)	23 (9.54%)	0.028	44 (9.36%)	23 (9.79%)	21 (8.94%)	0.029
Subdural_hematoma (YES), *n* (%)	1503 (63.20%)	1362 (63.73%)	141 (58.51%)	0.107	281 (59.79%)	141 (60.00%)	140 (59.57%)	0.009
Diffuse_brain_injury (YES), *n* (%)	**94 (3.95%)**	**74 (3.46%)**	**20 (8.30%)**	**0.207**	31 (6.60%)	14 (5.96%)	17 (7.23%)	0.051
APSIII	37.41 (16.35)	37.47 (16.52)	36.95 (14.80)	0.033	37.44 (15.00)	37.68 (15.15)	37.21 (14.88)	0.031
SOFA_score	**3.44 (2.45)**	**3.37 (2.42)**	**4.06 (2.62)**	**0.276**	4.12 (2.69)	4.16 (2.73)	4.08 (2.65)	0.03
Charlson	**3.33 (2.76)**	**3.40 (2.76)**	**2.71 (2.66)**	**0.257**	2.67 (2.80)	2.58 (2.93)	2.76 (2.67)	0.064
**Interventions (boolean for 1st 24 h)**								
Mechanical_ventilation_use (YES), *n* (%)	**1090 (45.84%)**	**891 (41.69%)**	**199 (82.57%)**	**0.929**	390 (82.98%)	197 (83.83%)	193 (82.13%)	0.045
Vasopressor_use (YES), *n* (%)	**332 (13.96%)**	**272 (12.73%)**	**60 (24.90%)**	**0.315**	123 (26.17%)	65 (27.66%)	58 (24.68%)	0.068
Midazolam_use (YES), *n* (%)	**217 (9.13%)**	**185 (8.66%)**	**32 (13.28%)**	**0.148**	63 (13.40%)	31 (13.19%)	32 (13.62%)	0.012
Propofol_use (YES), *n* (%)	**944 (39.70%)**	**736 (34.44%)**	**208 (86.31%)**	**1.25**	405 (86.17%)	203 (86.38%)	202 (85.96%)	0.012
Fentanyl_use (YES), *n* (%)	**777 (32.67%)**	**611 (28.59%)**	**166 (68.88%)**	**0.881**	325 (69.15%)	165 (70.21%)	160 (68.09%)	0.046
**Comorbidities (boolean)**								
HF (YES), *n* (%)	246 (10.34%)	228 (10.67%)	18 (7.47%)	0.112	33 (7.02%)	15 (6.38%)	18 (7.66%)	0.05
Renal (YES), *n* (%)	220 (9.25%)	203 (9.50%)	17 (7.05%)	0.089	33 (7.02%)	16 (6.81%)	17 (7.23%)	0.017
COPD (YES), *n* (%)	151 (6.35%)	138 (6.46%)	13 (5.39%)	0.045	30 (6.38%)	17 (7.23%)	13 (5.53%)	0.07
Stroke (YES), *n* (%)	162 (6.81%)	146 (6.83%)	16 (6.64%)	0.008	30 (6.38%)	14 (5.96%)	16 (6.81%)	0.035
Malignancy (YES), *n* (%)	22 (0.93%)	21 (0.98%)	1 (0.41%)	0.068	2 (0.43%)	1 (0.43%)	1 (0.43%)	<0.001
**Vital signs (1st 24 h)**								
MAP, mmHg	88.15 (17.05)	87.94 (16.98)	90.03 (17.63)	0.121	89.69 (17.80)	89.75 (17.97)	89.64 (17.67)	0.006
Heart rate, beats/min	84.77 (18.67)	84.61 (18.59)	86.14 (19.35)	0.08	86.44 (19.19)	87.12 (19.00)	85.75 (19.39)	0.072
Temperature, °C	**36.78 (0.87)**	**36.76 (0.88)**	**36.95 (0.77)**	**0.224**	36.88 (0.81)	36.84 (0.85)	36.92 (0.76)	0.096
SPO_2_, %	**97.69 (3.40)**	**97.58 (3.47)**	**98.64 (2.48)**	**0.351**	98.57 (2.94)	98.51 (3.33)	98.64 (2.50)	0.043
GCS	13.05 (2.65)	13.05 (2.66)	13.13 (2.56)	0.033	13.07 (2.74)	13.06 (2.89)	13.09 (2.58)	0.008
**Laboratory tests (1st 24 h)**								
WBC, 10^9/L	11.42 (6.50)	11.41 (6.67)	11.58 (4.79)	0.03	11.87 (4.67)	12.05 (4.56)	11.69 (4.79)	0.076
Hemoglobin, g/dl	11.64 (1.99)	11.63 (1.99)	11.76 (1.99)	0.065	11.82 (1.92)	11.88 (1.82)	11.77 (2.00)	0.056
Platelet, 10^9/L	**198.81 (75.56)**	**200.24 (76.64)**	**186.18 (63.90)**	**0.199**	186.62 (62.61)	185.60 (61.37)	187.64 (63.94)	0.033
Glucose, mg/dl	**141.68 (71.85)**	**142.21 (71.07)**	**136.93 (78.45)**	**0.071**	135.82 (66.01)	133.97 (49.37)	137.67 (79.30)	0.056
Sodium, mmol/l	138.79 (4.72)	138.79 (4.72)	138.86 (4.67)	0.015	138.76 (4.80)	138.59 (4.93)	138.93 (4.68)	0.071
Potassium, mmol/l	4.06 (0.69)	4.05 (0.69)	4.09 (0.67)	0.056	4.10 (0.70)	4.11 (0.73)	4.09 (0.67)	0.022
Bicarbonate, mmol/l	**23.08 (3.65)**	**23.18 (3.60)**	**22.19 (3.96)**	**0.263**	22.09 (3.81)	22.07 (3.66)	22.11 (3.96)	0.01
Chloride, mmol/l	104.01 (5.55)	104.03 (5.54)	103.83 (5.63)	0.037	103.95 (5.76)	103.98 (5.93)	103.92 (5.60)	0.01
BUN, mg/dl	**17.67 (12.27)**	**17.90 (12.42)**	**15.60 (10.63)**	**0.2**	15.37 (9.57)	14.92 (8.32)	15.83 (10.67)	0.095
Lactate, mmol/l	1.02 (0.89)	1.02 (0.89)	1.00 (0.86)	0.026	0.99 (0.76)	0.96 (0.63)	1.01 (0.87)	0.062
INR	**1.20 (0.35)**	**1.21 (0.37)**	**1.15 (0.15)**	**0.232**	1.15 (0.16)	1.14 (0.16)	1.15 (0.15)	0.049
PT, s	**13.30 (4.81)**	**13.40 (5.04)**	**12.43 (1.67)**	**0.259**	12.45 (1.70)	12.43 (1.74)	12.48 (1.66)	0.029
APTT, s	**28.81 (8.74)**	**28.95 (9.01)**	**27.57 (5.77)**	**0.182**	27.42 (5.39)	27.26 (4.90)	27.58 (5.84)	0.058
**Clinical outcome events**								
Cerebral surgery, *n* (%)	**1913 (80.45%)**	**1681 (78.66%)**	**232 (96.27%)**	**0.551**	453 (96.38%)	227 (96.60%)	226 (96.17%)	0.023
Sepsis, *n* (%)	**1022 (42.98%)**	**867 (40.57%)**	**155 (64.32%)**	**0.489**	297 (63.19%)	148 (62.98%)	149 (63.40%)	0.009
VAP, *n* (%)	**159 (6.69%)**	**123 (5.76%)**	**36 (14.94%)**	**0.305**	68 (14.47%)	35 (14.89%)	33 (14.04%)	0.024
**Mortality**								
28‐day_mortality, *n* (%)	**391 (16.44%)**	**364 (17.03%)**	**27 (11.20%)**	**0.168**	67 (14.26%)	40 (17.02%)	27 (11.49%)	0.159
Hosipital_mortality, *n* (%)	**291 (12.24%)**	**274 (12.82%)**	**17 (7.05%)**	**0.194**	**52 (11.06%)**	**35 (14.89%)**	**17 (7.23%)**	**0.246**
1‐year_mortality, *n* (%)	614 (25.82%)	564 (26.39%)	50 (20.75%)	0.133	108 (22.98%)	58 (24.68%)	50 (21.28%)	0.081

*Note*: Values are presented as mean (standard deviation) for continuous variables and number (percentage) for categorical variables. Variables in bold have *p*‐value < 0.05.

Abbreviations: APS III, acute physiology score III; APTT, activated partial thromboplastin time; BUN, blood urea nitrogen; COPD, chronic obstructive pulmonary disease; DEX, dexmedetomidine; GCS, Glasgow Coma Scale; HF, heart failure; INR, international normalized ratio; MAP, mean arterial pressure; PT, prothrombin time; SMD, standardized mean differences; SOFA, sequential organ failure assessment; SPO2, oxygen saturation; VAP, ventilator‐associated pneumonia; WBC, white blood cells.

**Figure 2:**
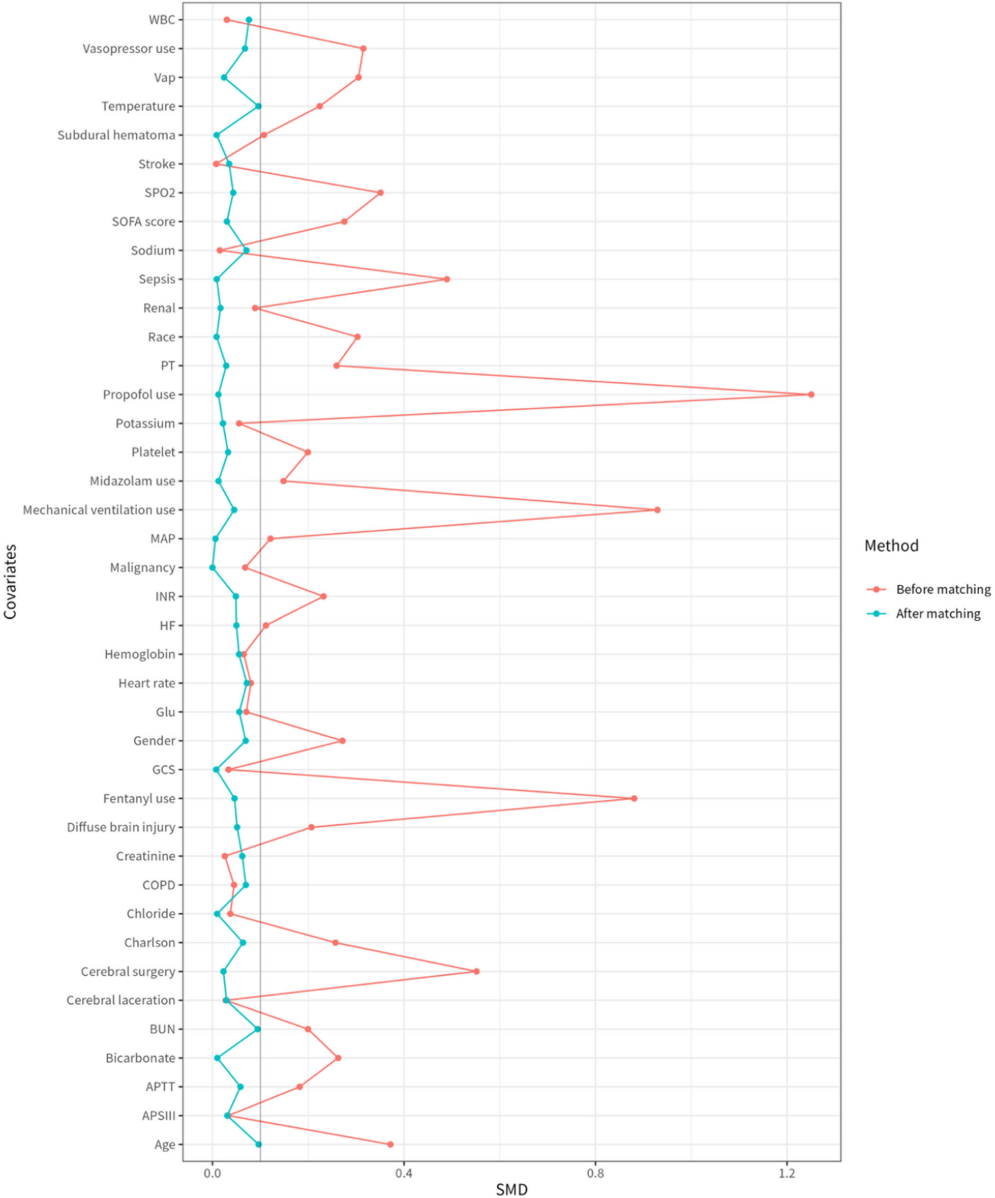
Changes in standardized mean difference (SMD) of cohort. APTT, activated partial thromboplastin time; APS III, acute physiology score III; BUN, blood urea nitrogen; COPD, chronic obstructive pulmonary disease; GCS, Glasgow Coma Scale; HF, heart failure; INR, international normalized ratio; MAP, mean arterial pressure; PT, prothrombin time; SPO2, oxygen saturation; SOFA, sequential organ failure assessment; SMD, standardized mean difference; VAP, ventilator‐associated pneumonia; WBC, white blood cells.

After PSM, the matched cohort consisted of 470 TBI patients, 235 for each group. All variables showed no significant differences (Supplementary Table 2). All covariates achieved SMD < 0.1 after PSM, confirming adequate balance (detailed in Supplementary Table 3 and Figure 2).

### 3.2 Association between DEX and primary and secondary outcomes

**Figure 3:**
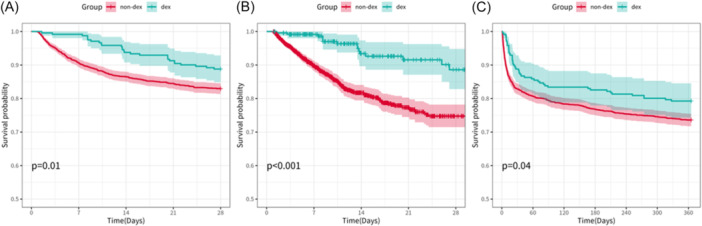
Unadjusted Kaplan–Meier survival curve for short‐ and long‐term mortality. (A) 28‐day mortality, (B) Hospital mortality, and (C) 365‐day mortality.

Unadjusted log‐rank tests and Kaplan‐Meier survival curves were performed on the original cohort to assess mortality outcomes (Table 2 and Supplementary Tables 4–6, Figure 3). For 28‐day mortality, the DEX group showed a significantly lower hazard compared to the non‐DEX group (HR 0.62, 95% CI 0.45–0.85, *p* < 0.05). For hospital mortality, the DEX group exhibited a markedly reduced hazard (HR 0.33, 95% CI 0.24–0.46, *p* < 0.001). Additionally, for 1‐year mortality, the DEX group demonstrated a reduced hazard (HR 0.74, 95% CI 0.57–0.95, *p* < 0.05).

**Table 2:** Primary and secondary outcome analyses with different models for cohort.

		28‐Day mortality		Hospital mortality		1‐year mortality	
Model	Group	*p*‐value	Result	*p*‐value	Result	*p*‐value	Result
Model 1^a^	non‐DEX		1 (Reference)		1 (Reference)		1 (Reference)
	DEX	**<0.05**	**0.62 (0.45, 0.85)**	**<0.001**	**0.33 (0.24, 0.46)**	**<0.05**	**0.74 (0.57, 0.95)**
Model 2^a^	non‐DEX		1 (Reference)		1 (Reference)		1 (Reference)
	DEX	**<0.001**	**0.45 (0.30, 0.69)**	**<0.001**	**0.25 (0.15, 0.42)**	**<0.01**	**0.64 (0.47, 0.87)**
Model 3^a^	non‐DEX		1 (Reference)		1 (Reference)		1 (Reference)
	DEX	**<0.001**	**0.48 (0.32, 0.72)**	**<0.001**	**0.28 (0.17, 0.46)**	**<0.05**	**0.67 (0.50, 0.92)**
Model 4^a^	non‐DEX		1 (Reference)		1 (Reference)		1 (Reference)
	DEX	**<0.001**	**0.46 (0.31, 0.70)**	**<0.001**	**0.25 (0.15, 0.43)**	**<0.01**	**0.64 (0.47, 0.88)**
Model 5^a^	non‐DEX		1 (Reference)		1 (Reference)		1 (Reference)
	DEX	**<0.05**	**0.57 (0.33, 0.96)**	**<0.001**	**0.20 (0.11, 0.37)**	0.08	0.72 (0.50, 1.04)

*Note*: Statistical analyses of different models with *p*‐value < 0.05 were displayed in bold.

Model 1: Log rank test [HR (95% CI)];

Model 2: Cox model adjusted with all covariates [HR (95% CI)];

Model 3: Cox model adjusted with unbalanced covariates [HR (95% CI)];

Model 4: Cox model adjusted with covariates selected by Boruta [HR (95% CI)];

Model 5: Cox proportional hazards model weighted by IPTW [HR (95% CI)].

^a^ HR = Hazard Ratio, CI = Confidence Interval.

**Figure 4:**
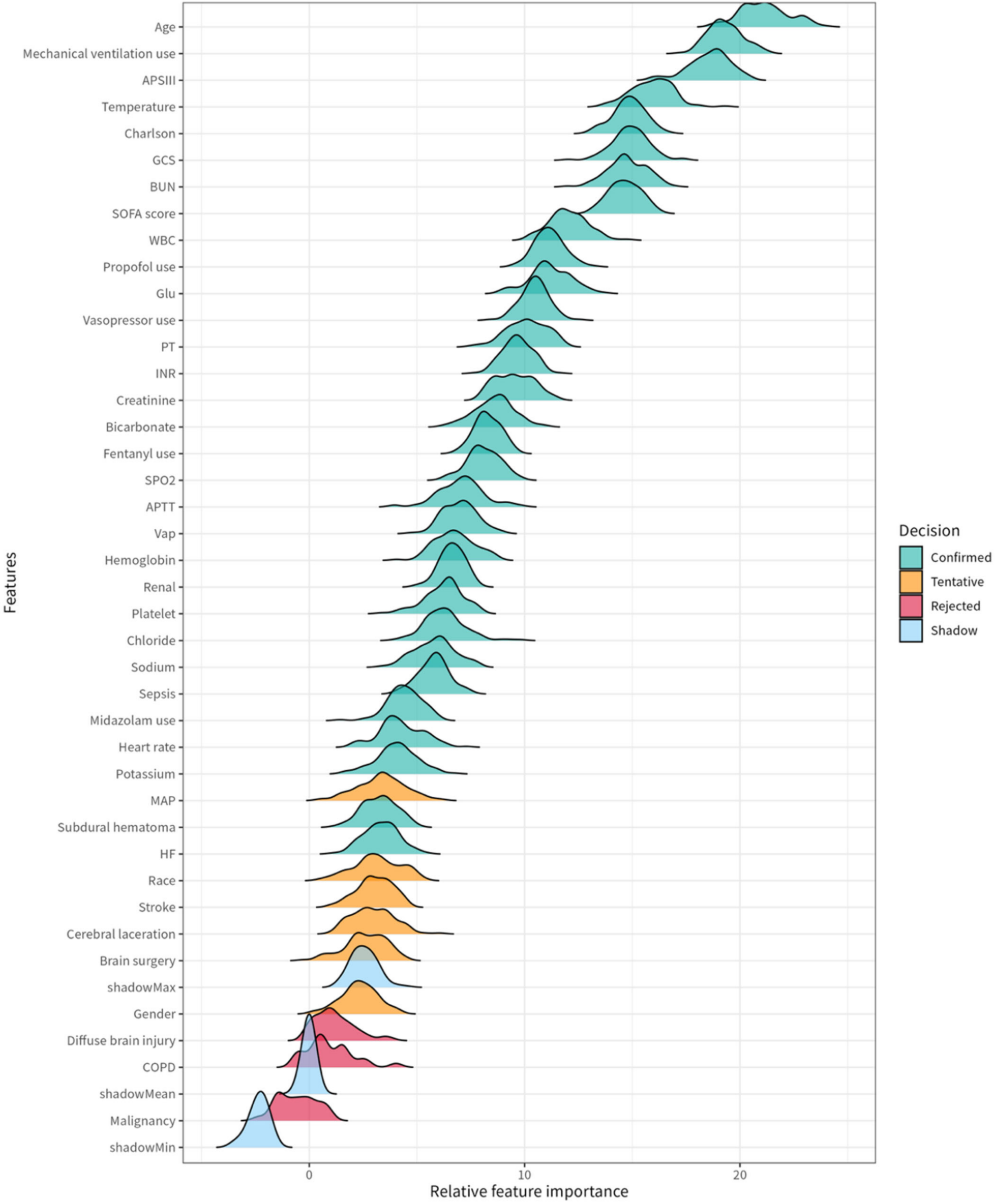
Importance of each variable according to the Boruta feature selection of cohort. APS III, acute physiology score III; APTT, activated partial thromboplastin time; BUN, blood urea nitrogen; COPD, chronic obstructive pulmonary disease; GCS, Glasgow Coma Scale; HF, heart failure; INR, international normalized ratio; MAP, mean arterial pressure; PT, prothrombin time; SOFA, sequential organ failure assessment; SPO2, oxygen saturation; VAP, ventilator‐associated pneumonia; WBC, white blood cells.

The association between early DEX use and 28‐day mortality was evaluated using Cox proportional hazards models, as summarized in Table 2. In the multivariable model adjusting for all covariates, DEX use was significantly associated with a reduced risk of 28‐day mortality (HR 0.45, 95% CI 0.30–0.69, *p* < 0.001). Similarly, in the multivariable model adjusting for unbalanced covariates—including age, gender, race, subdural hematoma, diffuse brain injury, SOFA score, Charlson Comorbidity Index, mechanical ventilation use, vasopressor use, midazolam use, propofol use, fentanyl use, HF, MAP, temperature, SpO_2_, platelets, bicarbonate, BUN, INR, PT, APTT, cerebral surgery, sepsis, and VAP—DEX remained protective (HR 0.48, 95% CI 0.32–0.72, *p* < 0.001). Furthermore, in the multivariable model adjusting for covariates selected through the Boruta feature selection, including VAP, sepsis, APTT, PT, INR, creatinine, BUN, chloride, bicarbonate, potassium, sodium, glucose, platelets, hemoglobin, WBC, GCS, SpO_2_, temperature, heart rate, renal disease, HF, fentanyl use, propofol use, midazolam use, vasopressor use, mechanical ventilation use, Charlson Comorbidity Index, SOFA score, APS III, subdural hematoma, and age, DEX continued to show protective effects (HR 0.46, 95% CI 0.31–0.70, *p* < 0.001) (Figure 4). This association was further corroborated by IPTW, which yielded a hazard ratio of 0.57 (95% CI 0.33–0.96, *p* < 0.05) (detailed in Supplementary Tables 7–10).

Secondary outcomes are detailed in Table 2. For hospital mortality, the multivariable model adjusting for all covariates showed a strong protective association with early DEX use (HR 0.25, 95% CI 0.15–0.42, *p* < 0.001). This effect remained robust in the multivariable model adjusting for unbalanced covariates (HR 0.28, 95% CI 0.17–0.46, *p* < 0.001) and the model adjusting for covariates selected by Boruta (HR 0.25, 95% CI 0.15–0.43, *p* < 0.001). IPTW further corroborated this effect, yielding an HR of 0.20 (95% CI 0.11–0.37, *p* < 0.001). For 1‐year mortality, early DEX use was associated with a reduced risk in the multivariable model adjusting for all covariates (HR 0.64, 95% CI 0.47–0.87, *p* < 0.01). This association held in the multivariable model adjusting for unbalanced covariates (HR 0.67, 95% CI 0.50–0.92, *p* < 0.05) and the Boruta model (HR 0.64, 95% CI 0.47–0.88, *p* < 0.01). However, the IPTW analysis indicated a trend toward reduced risk, although statistical significance was not reached (HR 0.72, 95% CI 0.50–1.04, *p* = 0.08) (detailed in Supplementary Tables 11–18).

### 3.3 Subgroup analysis in TBI patients

**Figure 5:**
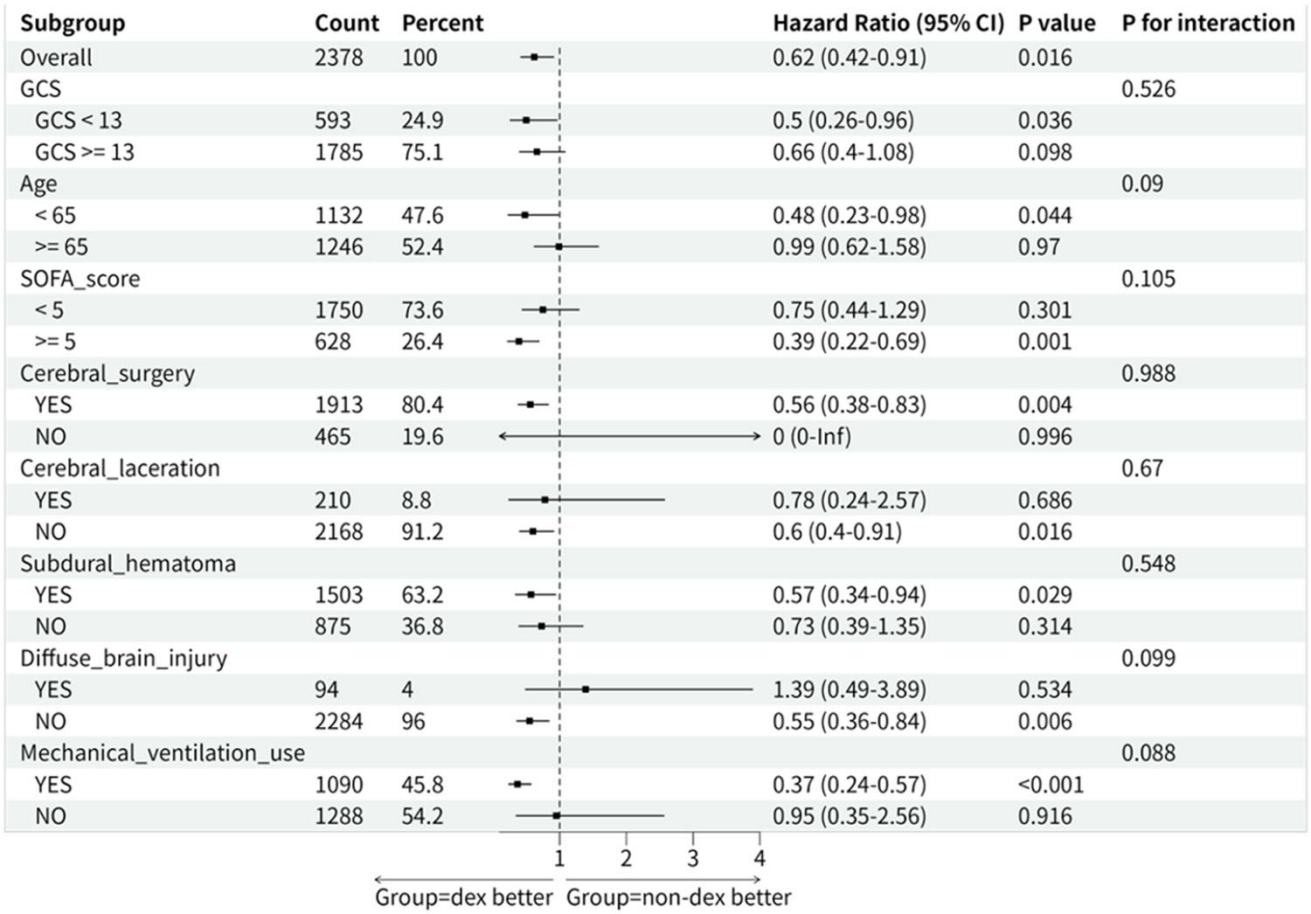
Forest plot of subgroup analysis for 28‐day mortality. GCS, Glasgow Coma Scale; SOFA, sequential organ failure assessment.

Stratified analyses were conducted across patient subgroups defined by age, GCS score, SOFA score, cerebral surgery, specific injury types, and mechanical ventilation use, aiming to investigate whether the association between early DEX use and 28‐day mortality varied among populations with different characteristics. Although tests for interaction were not statistically significant (all *p*‐value for interaction > 0.05, with trends noted for age and mechanical ventilation at *p* < 0.1), the point estimates for the hazard ratios suggested that the association between DEX and reduced mortality was significant in subgroups such as patients with younger age (<65 years), or those requiring mechanical ventilation. The robustness of the association between early DEX use and improved survival was supported across these subgroups (Figure 5).

## 4 DISCUSSION

This retrospective cohort study demonstrates that early initiation of DEX within 48 h of ICU admission is associated with robust survival benefits in patients with TBI, specifically reducing 28‐day and hospital mortality. Regarding long‐term prognosis, multivariable models consistently suggested a sustained benefit at 1 year. Notably, subgroup analyses suggested a trend toward greater survival benefit in younger patients (<65 years) and those requiring mechanical ventilation.

These observed short‐term survival benefits align with existing literature. Liu et al. analyzed data from the Premier Healthcare Database (PHD) and reported reduced hospital mortality in mechanically ventilated TBI patients receiving early DEX.^12^ Similarly, Xu et al. found that DEX reduced hospital mortality in TBI patients.^11^ These findings suggest that DEX administration can improve short‐term outcomes in TBI patients. Based on these results, the short‐term survival benefits observed with early DEX administration may be attributed to its neuroprotective effects during the acute phase of TBI.

The observed survival advantage likely stems from DEX's capacity to mitigate secondary brain injury, a complex cascade that evolves after the primary insult and is a critical determinant of TBI prognosis. Unlike the immediate damage caused by primary injury, secondary brain injury evolves over time through a cascade of physiological and biochemical processes that exacerbate the initial damage.^14^ Key contributors to secondary injury include elevated intracranial pressure (ICP), reduced cerebral perfusion pressure (CPP), and neuroinflammation—each of which can lead to further neuronal damage and functional decline.^15^, ^16^ Early DEX administration might improve outcomes in TBI patients requiring ICP monitoring.^17^ DEX use may help reduce ICP by decreasing cerebral blood volume, potentially through its effects on modulating cerebral vasculature.^18^ Furthermore, several animal models of TBI have demonstrated that DEX offers neuroprotective benefits, potentially through its ability to modulate neuroinflammation, autonomic dysfunction, and reduce sympathetic hyperactivity.DEX attenuates early brain injury by suppressing the NLRP3 inflammasome and reducing the infiltration of peripheral immune cells, ultimately promoting neurological recovery.^19^, ^20^ TBI‐induced autonomic dysfunction and inflammation can contribute to extracranial organ failure, further worsening overall outcomes. DEX may mitigate this by modulating neuroinflammation and the excessive sympathetic response, thereby preserving brain function and preventing additional secondary insults to the injured brain.^19^, ^20^, ^21^ The combined effects of neuroprotection and systemic stabilization likely explain the observed short‐term survival benefits.

Regarding long‐term prognosis, our study points to a sustained survival benefit at 1 year, suggesting that the physiological protection provided in the acute phase may translate into lasting effects. Although the signal for long‐term benefit was not as statistically overwhelming as that for short‐term mortality, the consistent trend toward risk reduction supports a potential enduring advantage. This contrasts with some previous reports, such as Liu et al.,^17^ which focused on moderate‐to‐severe TBI and did not identify a significant extended survival benefit in the entire population. This discrepancy is likely attributable to the heterogeneity in baseline injury severity between cohorts. Our population had a median GCS of 13, representing a milder injury spectrum compared to the severe cases in previous studies. In moderate‐to‐severe TBI, long‐term prognosis is often dominated by the irreversible structural damage of the primary injury, potentially creating a “therapeutic ceiling” that limits the long‐term impact of acute pharmacological interventions. Conversely, in patients with milder initial injuries (as in our cohort), mortality is frequently driven by potentially preventable secondary complications—such as progressive neuroinflammation, autonomic dysregulation, and delirium—rather than the primary neurological insult. Therefore, DEX's ability to attenuate these secondary insults may be more effective in this specific population, preventing the transition from mild injury to fatal deterioration and thereby securing long‐term survival.

Subgroup analyses suggested distinct trends toward greater survival benefits in two specific patient populations: younger patients (<65 years) and those requiring mechanical ventilation. Although tests for interaction did not reach statistical significance (0.05 < *p* < 0.1), the substantial reduction in hazard ratios within these specific subgroups warrants mechanistic consideration.

Regarding the age‐stratified analysis, the observation that younger patients derived greater benefit compared to older adults may be explained by age‐related differences in autonomic physiology. Younger TBI patients typically mount a robust, hyper‐adrenergic response to severe injury—often termed “sympathetic storming”—characterized by surges in circulating catecholamines that drive secondary systemic damage.^22^ DEX, by centrally suppressing sympathetic outflow, directly targets this pathological surge.^23^ In contrast, mortality in elderly patients is frequently driven by pre‐existing comorbidities (e.g., cardiovascular disease, frailty) and age‐related autonomic decline,^24^ which render the specific sympatholytic mechanism of DEX less impactful on their overall survival trajectory.

The survival benefit observed in patients requiring mechanical ventilation may be attributed to the unique pharmacological profile of DEX, which confers distinct physiological and clinical advantages in this high‐risk cohort. First, regarding physiological modulation, severe TBI often triggers autonomic dysregulation (sympathetic storming), leading to excessive catecholamine release and systemic inflammation. This creates a “double‐hit” injury when combined with the inflammatory stress of mechanical ventilation. DEX, a highly selective α‐2 adrenergic receptor agonist, interrupts this cascade by suppressing central sympathetic outflow.^25^ As a result, it reduces pro‐inflammatory cytokines (e.g., interleukin[IL]‐6, IL‐8) and attenuates the systemic inflammatory response.^26^ This mechanism not only mitigates secondary brain injury but also alleviates extracranial organ damage, particularly reducing ventilator‐induced lung injury and improving pulmonary compliance.^27^

Second, regarding sedation quality and respiratory management, DEX offers “cooperative sedation,” a state akin to natural sleep from which patients can be easily aroused (rousable sedation). Unlike GABA‐ergic agents (e.g., propofol or benzodiazepines) that depress respiratory drive, DEX preserves spontaneous breathing.^12^, ^16^, ^25^ This preservation maintains diaphragmatic tone, improves ventilator synchrony, and facilitates spontaneous breathing trials. Furthermore, the ability to wake patients easily allows for frequent neurological assessments without interrupting sedation, which is critical for monitoring TBI progression. Finally, DEX's established anti‐delirium properties facilitate smoother weaning processes and prevent agitation‐related adverse events.^28^, ^29^

Our findings shed light on the importance of early sedation strategies in critically ill TBI patients. Sedation plays a pivotal role in managing these patients by facilitating pain control, minimizing agitation, and reducing secondary brain injury. However, current sedation guidelines for TBI patients are lacking, creating a significant gap in clinical practice. This gap highlights the urgent need for evidence‐based sedation strategies that are personalized to the unique clinical needs of TBI patients. At present, DEX is often used as a secondary sedative agent rather than a first‐choice option. However, limited evidence has emerged showing DEX's ability to improve outcomes in TBI patients, such as reducing the duration of mechanical ventilation, ICU delirium, and inflammation. Our study expands the current evidence landscape, demonstrating that early DEX administration confers a distinct survival advantage in critically ill TBI patients, most notably in younger individuals (<65 years) and those requiring mechanical ventilation. These findings advocate for a targeted clinical approach: by prioritizing early DEX use in these specific subgroups, clinicians may better leverage its pharmacological benefits—such as reduced neuroinflammation, prevention of autonomic dysfunction, and improved ventilator synchrony—to mitigate multi‐organ dysfunction syndrome. Ultimately, this targeted strategy may translate into improved survival prognosis. However, given the retrospective nature of our analysis, further large‐scale randomized controlled trials are warranted to validate these findings and establish definitive clinical guidelines.

Several limitations should be considered when interpreting the findings of this study. First, the relatively small sample size in the DEX group may limit the robustness of our conclusions. Despite using PSM to reduce confounding, residual confounding factors may persist, and the exclusion of certain samples due to PSM may introduce bias, limiting the generalizability of the findings. Second, the low mortality rate in the DEX group restricted our ability to assess optimal timing and dosing, hindering a comprehensive understanding of DEX's therapeutic potential in TBI management. Third, being a retrospective study, it is subject to data incompleteness, selection bias, and unmeasured confounders. The reliance on previously collected clinical data, which may not have been consistently recorded or influenced by varying clinical practices, limits our ability to establish causality and may introduce bias into the results. Fourth, as a single‐center study, the external validity and generalizability of our findings are limited, as practices and outcomes may differ across institutions with varying protocols. Finally, although we aimed to identify TBI patients using ICD coding, excluding only those with concussions, misclassification, or bias in patient identification could still affect the accuracy of the population studied and introduce bias in the interpretation. Overall, further prospective studies are required to confirm and strengthen the robustness of our findings.

## 5 CONCLUSIONS

In this retrospective study, early administration of DEX (within 48 h of ICU admission) was associated with improved short‐ and long‐term survival in critically ill TBI patients. Notably, this survival benefit appeared particularly pronounced in younger patients and those requiring mechanical ventilation. These findings suggest that early DEX use may be a valuable neuroprotective strategy in this population. However, RCTs are essential to confirm these findings and establish causal relationships to guide clinical decision‐making.

## AUTHOR CONTRIBUTIONS

Yang Long, Long‐Zhen Liu, and Xiao‐Xue Tan contributed to analysis, picture production, and manuscript drafts. Yong‐Hu Zhang and Cheng‐Xi Liu are responsible for data collection and analysis. Shan Liu and Wen‐Jie Liu contributed to essential inputs and critical revisions. All authors contributed to the article and approved the submitted version.

## CONFLICT OF INTEREST STATEMENT

The authors declare no conflicts of interest.

## ETHICS STATEMENT

The MIMIC‐IV database was approved by the IRB of the Beth Israel Deaconess Medical Center and MIT. All data in the database were de‐identified to protect patient privacy. Therefore, this retrospective study did not require additional ethical approval or informed consent.

## Supporting information

Supporting information.

## Data Availability

The datasets presented in the current study are available in the MIMIC‐IV database. (https://physionet.org/content/mimiciv/3.1/).
