# Supplementary material for: Impact of early dexmedetomidine administration on short‐ and long‐term outcomes in critically traumatic brain injury patients: A retrospective study using the MIMIC‐IV database
Source: Ibrain. 2026 Mar 11;12(1):137–50. doi: 10.1002/ibra.70015 (PMC13097420; doi:10.1002/ibra.70015)
Supplement: Supplementary file 1 — Supporting information. [file IBRA-12-137-s001.docx]

Supplementary Table 1. Baseline characteristics before propensity score matching of cohort

|  | **Overall (N=2378)** | **non-DEX (N=2137)** | **DEX (N=241)** | ***p*-value** | **SMD** | **Missing data (%)** |
| --- | --- | --- | --- | --- | --- | --- |
| Age, years | **62.39 (21.79)** | **63.18 (21.83)** | **55.38 (20.11)** | **<0.001** | **0.372** | **0** |
| Gender (Female), n (%) | **869 (36.54%)** | **808 (37.81%)** | **61 (25.31%)** | **<0.001** | **0.271** | **0** |
| Race (White), n (%) | **925 (38.90%)** | **799 (37.39%)** | **126 (52.28%)** | **<0.001** | **0.303** | **0** |
| Cerebral laceration (YES), n (%) | 210 (8.83%) | 187 (8.75%) | 23 (9.54%) | 0.77 | 0.028 | 0 |
| Subdural hematoma (YES), n (%) | 1503 (63.20%) | 1362 (63.73%) | 141 (58.51%) | 0.13 | 0.107 | 0 |
| Diffuse brain injury (YES), n (%) | **94 (3.95%)** | **74 (3.46%)** | **20 (8.30%)** | **<0.001** | **0.207** | **0** |
| APSIII | 37.41 (16.35) | 37.47 (16.52) | 36.95 (14.80) | 0.98 | 0.033 | 0 |
| SOFA score | **3.44 (2.45)** | **3.37 (2.42)** | **4.06 (2.62)** | **<0.001** | **0.276** | **0** |
| Charlson | **3.33 (2.76)** | **3.40 (2.76)** | **2.71 (2.66)** | **<0.001** | **0.257** | **0** |
| **Interventions (boolean for 1st 24 h)** |  |  |  |  |  |  |
| Mechanical ventilation use (YES), n (%) | **1090 (45.84%)** | **891 (41.69%)** | **199 (82.57%)** | **<0.001** | **0.929** | **0** |
| Vasopressor use (YES), n (%) | **332 (13.96%)** | **272 (12.73%)** | **60 (24.90%)** | **<0.001** | **0.315** | **0** |
| Midazolam use (YES), n (%) | **217 (9.13%)** | **185 (8.66%)** | **32 (13.28%)** | **<0.05** | **0.148** | **0** |
| Propofol use (YES), n (%) | **944 (39.70%)** | **736 (34.44%)** | **208 (86.31%)** | **<0.001** | **1.25** | **0** |
| Fentanyl use (YES), n (%) | **777 (32.67%)** | **611 (28.59%)** | **166 (68.88%)** | **<0.001** | **0.881** | **0** |
| **Comorbidities (boolean)** |  |  |  |  |  |  |
| HF (YES), n (%) | 246 (10.34%) | 228 (10.67%) | 18 (7.47%) | 0.15 | 0.112 | 0 |
| Renal (YES), n (%) | 220 (9.25%) | 203 (9.50%) | 17 (7.05%) | 0.26 | 0.089 | 0 |
| COPD (YES), n (%) | 151 (6.35%) | 138 (6.46%) | 13 (5.39%) | 0.62 | 0.045 | 0 |
| Stroke (YES), n (%) | 162 (6.81%) | 146 (6.83%) | 16 (6.64%) | 1 | 0.008 | 0 |
| Malignancy (YES), n (%) | 22 (0.93%) | 21 (0.98%) | 1 (0.41%) | 0.6 | 0.068 | 0 |
| **Vital signs (1st 24 h)** |  |  |  |  |  |  |
| MAP, mmHg | 88.15 (17.05) | 87.94 (16.98) | 90.03 (17.63) | 0.08 | 0.121 | 0 |
| Heart rate, beats/min | 84.77 (18.67) | 84.61 (18.59) | 86.14 (19.35) | 0.43 | 0.08 | 0 |
| Temperature, ℃ | **36.78 (0.87)** | **36.76 (0.88)** | **36.95 (0.77)** | **<0.01** | **0.224** | **0** |
| SPO2, % | **97.69 (3.40)** | **97.58 (3.47)** | **98.64 (2.48)** | **<0.001** | **0.351** | **0** |
| GCS | 13.05 (2.65) | 13.05 (2.66) | 13.13 (2.56) | 0.05 | 0.033 | 0 |
| **Laboratory tests (1st 24 h)** |  |  |  |  |  |  |
| WBC, 10^9/L | 11.42 (6.50) | 11.41 (6.67) | 11.58 (4.79) | 0.17 | 0.03 | 0 |
| Hemoglobin, g/dl | 11.64 (1.99) | 11.63 (1.99) | 11.76 (1.99) | 0.3 | 0.065 | 0 |
| Platelet, 10^9/L | **198.81 (75.56)** | **200.24 (76.64)** | **186.18 (63.90)** | **<0.01** | **0.199** | **0** |
| Glucose, mg/dl | **141.68 (71.85)** | **142.21 (71.07)** | **136.93 (78.45)** | **<0.01** | **0.071** | **0** |
| Sodium, mmol/l | 138.79 (4.72) | 138.79 (4.72) | 138.86 (4.67) | 0.72 | 0.015 | 0 |
| Potassium, mmol/l | 4.06 (0.69) | 4.05 (0.69) | 4.09 (0.67) | 0.54 | 0.056 | 0 |
| Bicarbonate, mmol/l | **23.08 (3.65)** | **23.18 (3.60)** | **22.19 (3.96)** | **<0.001** | **0.263** | **0** |
| Chloride, mmol/l | 104.01 (5.55) | 104.03 (5.54) | 103.83 (5.63) | 0.94 | 0.037 | 0 |
| BUN, mg/dl | **17.67 (12.27)** | **17.90 (12.42)** | **15.60 (10.63)** | **<0.001** | **0.2** | **0** |
| Lactate, mmol/l | 1.02 (0.89) | 1.02 (0.89) | 1.00 (0.86) | 0.61 | 0.026 | 0 |
| INR | **1.20 (0.35)** | **1.21 (0.37)** | **1.15 (0.15)** | **<0.01** | **0.232** | **0** |
| PT, s | **13.30 (4.81)** | **13.40 (5.04)** | **12.43 (1.67)** | **<0.001** | **0.259** | **0** |
| APTT, s | **28.81 (8.74)** | **28.95 (9.01)** | **27.57 (5.77)** | **<0.01** | **0.182** | **0** |
| **Clinical outcome events** |  |  |  |  |  |  |
| Cerebral surgery, n (%) | **1913 (80.45%)** | **1681 (78.66%)** | **232 (96.27%)** | **<0.001** | **0.551** | **0** |
| Sepsis, n (%) | **1022 (42.98%)** | **867 (40.57%)** | **155 (64.32%)** | **<0.001** | **0.489** | **0** |
| Vap, n (%) | **159 (6.69%)** | **123 (5.76%)** | **36 (14.94%)** | **<0.001** | **0.305** | **0** |
| **Mortality** |  |  |  |  |  |  |
| 28-day mortality, n (%) | **391 (16.44%)** | **364 (17.03%)** | **27 (11.20%)** | **<0.05** | **0.168** | **0** |
| Hosipital mortality, n (%) | **291 (12.24%)** | **274 (12.82%)** | **17 (7.05%)** | **<0.05** | **0.194** | **0** |
| 1-year mortality, n (%) | 614 (25.82%) | 564 (26.39%) | 50 (20.75%) | 0.07 | 0.133 | 0 |
| Values are presented as mean (standard deviation) for continuous variables and number (percentage) for categorical variables. Variables in bold have *p*-value < 0.05.  Abbreviation: DEX, dexmedetomidine; SMD, standardized mean differences; APS III, acute physiology score III; SOFA, sequential organ failure assessment; HF, heart failure; COPD, chronic obstructive pulmonary disease; MAP, mean arterial pressure; SPO2, oxygen saturation; GCS, Glasgow Coma Scale; VAP, ventilator-associated pneumonia; WBC, white blood cells; BUN, blood urea nitrogen; INR, international normalized ratio; PT, prothrombin time; APTT, activated partial thromboplastin time. | | | | | | |

Supplementary Table 2. Baseline characteristics after propensity score matching of cohort

|  | **Overall (N=470)** | **non-DEX (N=235)** | **DEX (N=235)** | ***p*-value** | **SMD** | **Missing data (%)** |
| --- | --- | --- | --- | --- | --- | --- |
| Age, years | 54.87 (20.99) | 53.86 (21.87) | 55.89 (20.07) | 0.31 | 0.097 | 0 |
| Gender (Female), n (%) | 115 (24.47%) | 54 (22.98%) | 61 (25.96%) | 0.52 | 0.069 | 0 |
| Race (White), n (%) | 243 (51.70%) | 122 (51.91%) | 121 (51.49%) | 1 | 0.009 | 0 |
| Cerebral laceration (YES), n (%) | 44 (9.36%) | 23 (9.79%) | 21 (8.94%) | 0.87 | 0.029 | 0 |
| Subdural hematoma (YES), n (%) | 281 (59.79%) | 141 (60.00%) | 140 (59.57%) | 1 | 0.009 | 0 |
| Diffuse brain injury (YES), n (%) | 31 (6.60%) | 14 (5.96%) | 17 (7.23%) | 0.71 | 0.051 | 0 |
| APSIII | 37.44 (15.00) | 37.68 (15.15) | 37.21 (14.88) | 0.71 | 0.031 | 0 |
| SOFA score | 4.12 (2.69) | 4.16 (2.73) | 4.08 (2.65) | 0.84 | 0.03 | 0 |
| Charlson | 2.67 (2.80) | 2.58 (2.93) | 2.76 (2.67) | 0.22 | 0.064 | 0 |
| **Interventions (boolean for 1st 24 h)** |  |  |  |  |  |  |
| Mechanical ventilation use (YES), n (%) | 390 (82.98%) | 197 (83.83%) | 193 (82.13%) | 0.71 | 0.045 | 0 |
| Vasopressor use (YES), n (%) | 123 (26.17%) | 65 (27.66%) | 58 (24.68%) | 0.53 | 0.068 | 0 |
| Midazolam use (YES), n (%) | 63 (13.40%) | 31 (13.19%) | 32 (13.62%) | 1 | 0.012 | 0 |
| Propofol use (YES), n (%) | 405 (86.17%) | 203 (86.38%) | 202 (85.96%) | 1 | 0.012 | 0 |
| Fentanyl use (YES), n (%) | 325 (69.15%) | 165 (70.21%) | 160 (68.09%) | 0.69 | 0.046 | 0 |
| **Comorbidities (boolean)** |  |  |  |  |  |  |
| HF (YES), n (%) | 33 (7.02%) | 15 (6.38%) | 18 (7.66%) | 0.72 | 0.05 | 0 |
| Renal (YES), n (%) | 33 (7.02%) | 16 (6.81%) | 17 (7.23%) | 1 | 0.017 | 0 |
| COPD (YES), n (%) | 30 (6.38%) | 17 (7.23%) | 13 (5.53%) | 0.57 | 0.07 | 0 |
| Stroke (YES), n (%) | 30 (6.38%) | 14 (5.96%) | 16 (6.81%) | 0.85 | 0.035 | 0 |
| Malignancy (YES), n (%) | 2 (0.43%) | 1 (0.43%) | 1 (0.43%) | 1 | <0.001 | 0 |
| **Vital signs (1st 24 h)** |  |  |  |  |  |  |
| MAP, mmHg | 89.69 (17.80) | 89.75 (17.97) | 89.64 (17.67) | 0.72 | 0.006 | 0 |
| Heart rate, beats/min | 86.44 (19.19) | 87.12 (19.00) | 85.75 (19.39) | 0.18 | 0.072 | 0 |
| Temperature, ℃ | 36.88 (0.81) | 36.84 (0.85) | 36.92 (0.76) | 0.44 | 0.096 | 0 |
| SPO2, % | 98.57 (2.94) | 98.51 (3.33) | 98.64 (2.50) | 0.51 | 0.043 | 0 |
| GCS | 13.07 (2.74) | 13.06 (2.89) | 13.09 (2.58) | 0.93 | 0.008 | 0 |
| **Laboratory tests (1st 24 h)** |  |  |  |  |  |  |
| WBC, 10^9/L | 11.87 (4.67) | 12.05 (4.56) | 11.69 (4.79) | 0.19 | 0.076 | 0 |
| Hemoglobin, g/dl | 11.82 (1.92) | 11.88 (1.82) | 11.77 (2.00) | 0.55 | 0.056 | 0 |
| Platelet, 10^9/L | 186.62 (62.61) | 185.60 (61.37) | 187.64 (63.94) | 0.86 | 0.033 | 0 |
| Glucose, mg/dl | 135.82 (66.01) | 133.97 (49.37) | 137.67 (79.30) | 0.54 | 0.056 | 0 |
| Sodium, mmol/l | 138.76 (4.80) | 138.59 (4.93) | 138.93 (4.68) | 0.71 | 0.071 | 0 |
| Potassium, mmol/l | 4.10 (0.70) | 4.11 (0.73) | 4.09 (0.67) | 0.72 | 0.022 | 0 |
| Bicarbonate, mmol/l | 22.09 (3.81) | 22.07 (3.66) | 22.11 (3.96) | 0.92 | 0.01 | 0 |
| Chloride, mmol/l | 103.95 (5.76) | 103.98 (5.93) | 103.92 (5.60) | 0.69 | 0.01 | 0 |
| BUN, mg/dl | 15.37 (9.57) | 14.92 (8.32) | 15.83 (10.67) | 0.58 | 0.095 | 0 |
| Lactate, mmol/l | 0.99 (0.76) | 0.96 (0.63) | 1.01 (0.87) | 0.89 | 0.062 | 0 |
| INR | 1.15 (0.16) | 1.14 (0.16) | 1.15 (0.15) | 0.44 | 0.049 | 0 |
| PT, s | 12.45 (1.70) | 12.43 (1.74) | 12.48 (1.66) | 0.67 | 0.029 | 0 |
| APTT, s | 27.42 (5.39) | 27.26 (4.90) | 27.58 (5.84) | 0.32 | 0.058 | 0 |
| **Clinical outcome events** |  |  |  |  |  |  |
| Cerebral surgery, n (%) | 453 (96.38%) | 227 (96.60%) | 226 (96.17%) | 1 | 0.023 | 0 |
| Sepsis, n (%) | 297 (63.19%) | 148 (62.98%) | 149 (63.40%) | 1 | 0.009 | 0 |
| Vap, n (%) | 68 (14.47%) | 35 (14.89%) | 33 (14.04%) | 0.9 | 0.024 | 0 |
| **Mortality** |  |  |  |  |  |  |
| 28-day mortality, n (%) | 67 (14.26%) | 40 (17.02%) | 27 (11.49%) | 0.11 | 0.159 | 0 |
| Hosipital mortality, n (%) | **52 (11.06%)** | **35 (14.89%)** | **17 (7.23%)** | **<0.05** | **0.246** | **0** |
| 1-year mortality, n (%) | 108 (22.98%) | 58 (24.68%) | 50 (21.28%) | 0.44 | 0.081 | 0 |
| Values are presented as mean (standard deviation) for continuous variables and number (percentage) for categorical variables. Variables in bold have *p*-value < 0.05.  Abbreviation: DEX, dexmedetomidine; SMD, standardized mean differences; APS III, acute physiology score III; SOFA, sequential organ failure assessment; HF, heart failure; COPD, chronic obstructive pulmonary disease; MAP, mean arterial pressure; SPO2, oxygen saturation; GCS, Glasgow Coma Scale; VAP, ventilator-associated pneumonia; WBC, white blood cells; BUN, blood urea nitrogen; INR, international normalized ratio; PT, prothrombin time; APTT, activated partial thromboplastin time. | | | | | | |

Supplementary Table 3. SMD of covariates before and after propensity score matching of cohort

| Characteristic | Before matcing | After matcing |
| --- | --- | --- |
| SMD ≤ 0.1 | 15 | 40 |
| SMD > 0.1 | 25 | 0 |
| Total number of covariates | 40 | 40 |
| Abbreviation: SMD, standardized mean differences. | | |

Supplementary Table 4. Unadjusted log-rank test for 28-day mortality of original cohort

| Group | HR | 95% CI | *p-*value |
| --- | --- | --- | --- |
| non-DEX | 1 | — | — |
| DEX | 0.62 | 0.45, 0.85 | 0.01 |
| Abbreviations: CI, Confidence Interval; HR, Hazard Ratio; DEX, dexmedetomidine. | | | |

Supplementary Table 5. Unadjusted log-rank test for Hos mortality of original cohort

| Group | HR | 95% CI | *p-*value |
| --- | --- | --- | --- |
| non-DEX | 1 | — | — |
| DEX | 0.33 | 0.24, 0.46 | <0.001 |
| Abbreviations: CI, Confidence Interval; HR, Hazard Ratio; DEX, dexmedetomidine. | | | |
|  | | | |

Supplementary Table 6. Unadjusted log-rank test for 1-year mortality of original cohort

| Group | HR | 95% CI | *p-*value |
| --- | --- | --- | --- |
| non-DEX | 1 | — | — |
| DEX | 0.74 | 0.57, 0.95 | 0.04 |
| Abbreviations: CI, Confidence Interval; HR, Hazard Ratio; DEX, dexmedetomidine. | | | |

Supplementary Table 7. Multivariate Cox model adjusted with all covariates for 28-day mortality of original cohort

| **Characteristic** | **HR** | **95% CI** | ***p*-value** |
| --- | --- | --- | --- |
| Group |  |  |  |
| non-DEX | 1.00 | Reference |  |
| DEX | 0.45 | 0.30, 0.69 | <0.001 |
| Age | 1.03 | 1.02, 1.04 | <0.001 |
| Gender |  |  |  |
| Female | 1.00 | Reference |  |
| Male | 0.99 | 0.79, 1.23 | 0.91 |
| Race |  |  |  |
| White | 1.00 | Reference |  |
| other | 0.84 | 0.67, 1.04 | 0.11 |
| Cerebral laceration |  |  |  |
| YES | 1.00 | Reference |  |
| NO | 0.75 | 0.50, 1.11 | 0.15 |
| Subdural hematoma |  |  |  |
| YES | 1.00 | Reference |  |
| NO | 0.81 | 0.64, 1.03 | 0.09 |
| Diffuse brain injury |  |  |  |
| YES | 1.00 | Reference |  |
| NO | 0.74 | 0.45, 1.21 | 0.23 |
| APSIII | 1.02 | 1.01, 1.03 | <0.001 |
| SOFA score | 1.07 | 1.01, 1.13 | 0.02 |
| Charlson | 0.99 | 0.92, 1.07 | 0.85 |
| Mechanical ventilation use |  |  |  |
| YES | 1.00 | Reference |  |
| NO | 0.17 | 0.12, 0.24 | <0.001 |
| Vasopressor use |  |  |  |
| YES | 1.00 | Reference |  |
| NO | 0.63 | 0.47, 0.84 | 0.002 |
| Midazolam use |  |  |  |
| YES | 1.00 | Reference |  |
| NO | 2.21 | 1.43, 3.42 | <0.001 |
| Propofol use |  |  |  |
| YES | 1.00 | Reference |  |
| NO | 1.55 | 1.14, 2.11 | 0.006 |
| Fentanyl use |  |  |  |
| YES | 1.00 | Reference |  |
| NO | 1.45 | 1.07, 1.96 | 0.02 |
| HF |  |  |  |
| YES | 1.00 | Reference |  |
| NO | 1.28 | 0.93, 1.78 | 0.13 |
| Renal |  |  |  |
| YES | 1.00 | Reference |  |
| NO | 0.99 | 0.67, 1.45 | 0.96 |
| COPD |  |  |  |
| YES | 1.00 | Reference |  |
| NO | 0.86 | 0.57, 1.29 | 0.46 |
| Stroke |  |  |  |
| YES | 1.00 | Reference |  |
| NO | 0.71 | 0.51, 0.99 | 0.04 |
| Malignancy |  |  |  |
| YES | 1.00 | Reference |  |
| NO | 1.36 | 0.43, 4.28 | 0.6 |
| MAP | 1.01 | 1.00, 1.01 | 0.09 |
| Heart rate | 1.00 | 1.00, 1.01 | 0.52 |
| Temperature | 0.83 | 0.76, 0.91 | <0.001 |
| SPO2 | 1.00 | 0.97, 1.03 | 0.83 |
| GCS | 1.04 | 0.99, 1.08 | 0.11 |
| WBC | 1.01 | 1.00, 1.02 | 0.008 |
| Hemoglobin | 0.95 | 0.90, 1.01 | 0.08 |
| Platelet | 1.00 | 1.00, 1.00 | 0.83 |
| Glu | 1.00 | 1.00, 1.00 | 0.25 |
| Sodium | 1.04 | 1.01, 1.08 | 0.007 |
| Potassium | 1.24 | 1.08, 1.43 | 0.002 |
| Bicarbonate | 1.00 | 0.97, 1.03 | 0.95 |
| Chloride | 0.98 | 0.95, 1.01 | 0.2 |
| BUN | 1.01 | 1.00, 1.01 | 0.1 |
| Creatinine | 0.89 | 0.78, 1.03 | 0.13 |
| INR | 3.18 | 1.19, 8.54 | 0.02 |
| PT | 0.92 | 0.85, 1.00 | 0.06 |
| APTT | 1.00 | 0.99, 1.01 | 0.56 |
| Cerebral surgery |  |  |  |
| YES | 1.00 | Reference |  |
| NO | 0.84 | 0.58, 1.23 | 0.37 |
| Sepsis |  |  |  |
| YES | 1.00 | Reference |  |
| NO | 1.03 | 0.82, 1.29 | 0.82 |
| Vap |  |  |  |
| YES | 1.00 | Reference |  |
| NO | 2.21 | 1.44, 3.38 | <0.001 |
| Abbreviations: CI, Confidence Interval; HR, Hazard Ratio; DEX, dexmedetomidine; APS III, acute physiology score III; SOFA, sequential organ failure assessment; HF, heart failure; COPD, chronic obstructive pulmonary disease; MAP, mean arterial pressure; SPO2, oxygen saturation; GCS, Glasgow Coma Scale; VAP, ventilator-associated pneumonia; WBC, white blood cells; BUN, blood urea nitrogen; INR, international normalized ratio; PT, prothrombin time; APTT, activated partial thromboplastin time. | | | |

Supplementary Table 8. Multivariate Cox model adjusted with unbalanced covariates for 28-day mortality of cohort

| **Characteristic** | **HR** | **95% CI** | ***p-*value** |
| --- | --- | --- | --- |
| Group |  |  |  |
| non-DEX | 1.00 | Reference |  |
| DEX | 0.48 | 0.32, 0.72 | <0.001 |
| Age | 1.03 | 1.02, 1.04 | <0.001 |
| Gender |  |  |  |
| Female | 1.00 | Reference |  |
| Male | 0.87 | 0.71, 1.08 | 0.21 |
| Race |  |  |  |
| White | 1.00 | Reference |  |
| other | 0.90 | 0.73, 1.12 | 0.34 |
| Subdural hematoma |  |  |  |
| YES | 1.00 | Reference |  |
| NO | 0.87 | 0.70, 1.09 | 0.22 |
| Diffuse brain injury |  |  |  |
| YES | 1.00 | Reference |  |
| NO | 0.80 | 0.49, 1.29 | 0.36 |
| SOFA score | 1.15 | 1.10, 1.20 | <0.001 |
| Charlson | 1.04 | 0.98, 1.11 | 0.18 |
| Mechanical ventilation use |  |  |  |
| YES | 1.00 | Reference |  |
| NO | 0.15 | 0.10, 0.20 | <0.001 |
| Vasopressor use |  |  |  |
| YES | 1.00 | Reference |  |
| NO | 0.59 | 0.44, 0.78 | <0.001 |
| Midazolam use |  |  |  |
| YES | 1.00 | Reference |  |
| NO | 2.08 | 1.36, 3.17 | <0.001 |
| Propofol use |  |  |  |
| YES | 1.00 | Reference |  |
| NO | 2.06 | 1.53, 2.76 | <0.001 |
| Fentanyl use |  |  |  |
| YES | 1.00 | Reference |  |
| NO | 1.35 | 1.01, 1.81 | 0.05 |
| HF |  |  |  |
| YES | 1.00 | Reference |  |
| NO | 1.27 | 0.92, 1.76 | 0.14 |
| MAP | 1.00 | 1.00, 1.01 | 0.3 |
| Temperature | 0.79 | 0.73, 0.87 | <0.001 |
| SPO2 | 1.01 | 0.98, 1.04 | 0.63 |
| Platelet | 1.00 | 1.00, 1.00 | 0.32 |
| Bicarbonate | 1.00 | 0.97, 1.03 | 0.9 |
| BUN | 1.01 | 1.00, 1.02 | 0.004 |
| INR | 3.32 | 1.10, 10.0 | 0.03 |
| PT | 0.92 | 0.84, 1.01 | 0.09 |
| APTT | 1.00 | 0.99, 1.01 | 0.75 |
| Cerebral surgery |  |  |  |
| YES | 1.00 | Reference |  |
| NO | 0.89 | 0.61, 1.29 | 0.53 |
| Sepsis |  |  |  |
| YES | 1.00 | Reference |  |
| NO | 0.99 | 0.79, 1.24 | 0.94 |
| Vap |  |  |  |
| YES | 1.00 | Reference |  |
| NO | 1.86 | 1.23, 2.81 | 0.003 |
| Abbreviations: CI, Confidence Interval; HR, Hazard Ratio; DEX, dexmedetomidine; APS III, acute physiology score III; SOFA, sequential organ failure assessment; HF, heart failure; COPD, chronic obstructive pulmonary disease; MAP, mean arterial pressure; SPO2, oxygen saturation; GCS, Glasgow Coma Scale; VAP, ventilator-associated pneumonia; WBC, white blood cells; BUN, blood urea nitrogen; INR, international normalized ratio; PT, prothrombin time; APTT, activated partial thromboplastin time. | | | |

Supplementary Table 9. Multivariate Cox model adjusted with covariates selected by Boruta algorithm for 28-day mortality of cohort

| **Characteristic** | **HR** | **95% CI** | ***p-*value** |
| --- | --- | --- | --- |
| Group |  |  |  |
| non-DEX | 1.00 | Reference |  |
| DEX | 0.46 | 0.31, 0.70 | <0.001 |
| Vap |  |  |  |
| YES | 1.00 | Reference |  |
| NO | 2.15 | 1.41, 3.28 | <0.001 |
| Sepsis |  |  |  |
| YES | 1.00 | Reference |  |
| NO | 0.99 | 0.79, 1.25 | 0.95 |
| APTT | 1.00 | 0.99, 1.02 | 0.51 |
| PT | 0.92 | 0.84, 1.00 | 0.06 |
| INR | 3.44 | 1.20, 9.91 | 0.02 |
| Creatinine | 0.90 | 0.78, 1.04 | 0.17 |
| BUN | 1.01 | 1.00, 1.01 | 0.1 |
| Chloride | 0.98 | 0.96, 1.01 | 0.31 |
| Bicarbonate | 1.00 | 0.97, 1.03 | 0.88 |
| Potassium | 1.23 | 1.07, 1.41 | 0.003 |
| Sodium | 1.04 | 1.01, 1.07 | 0.02 |
| Glu | 1.00 | 1.00, 1.00 | 0.17 |
| Platelet | 1.00 | 1.00, 1.00 | 0.99 |
| Hemoglobin | 0.96 | 0.91, 1.01 | 0.13 |
| WBC | 1.01 | 1.00, 1.02 | 0.01 |
| GCS | 1.03 | 0.98, 1.08 | 0.2 |
| SPO2 | 1.00 | 0.97, 1.04 | 0.76 |
| Temperature | 0.84 | 0.77, 0.91 | <0.001 |
| Heart rate | 1.00 | 1.00, 1.01 | 0.32 |
| Renal |  |  |  |
| YES | 1.00 | Reference |  |
| NO | 0.99 | 0.68, 1.44 | 0.95 |
| HF |  |  |  |
| YES | 1.00 | Reference |  |
| NO | 1.28 | 0.93, 1.76 | 0.13 |
| Fentanyl use |  |  |  |
| YES | 1.00 | Reference |  |
| NO | 1.40 | 1.04, 1.89 | 0.03 |
| Propofol use |  |  |  |
| YES | 1.00 | Reference |  |
| NO | 1.61 | 1.18, 2.19 | 0.002 |
| Midazolam use |  |  |  |
| YES | 1.00 | Reference |  |
| NO | 2.34 | 1.52, 3.62 | <0.001 |
| Vasopressor use |  |  |  |
| YES | 1.00 | Reference |  |
| NO | 0.64 | 0.48, 0.86 | 0.003 |
| Mechanical ventilation use |  |  |  |
| YES | 1.00 | Reference |  |
| NO | 0.16 | 0.11, 0.22 | <0.001 |
| Charlson | 1.01 | 0.94, 1.09 | 0.78 |
| SOFA score | 1.07 | 1.01, 1.13 | 0.02 |
| APSIII | 1.02 | 1.01, 1.03 | <0.001 |
| Subdural hematoma |  |  |  |
| YES | 1.00 | Reference |  |
| NO | 0.86 | 0.69, 1.08 | 0.19 |
| Age | 1.03 | 1.02, 1.04 | <0.001 |
| Abbreviations: CI, Confidence Interval; HR, Hazard Ratio; DEX, dexmedetomidine; APS III, acute physiology score III; SOFA, sequential organ failure assessment; HF, heart failure; COPD, chronic obstructive pulmonary disease; MAP, mean arterial pressure; SPO2, oxygen saturation; GCS, Glasgow Coma Scale; VAP, ventilator-associated pneumonia; WBC, white blood cells; BUN, blood urea nitrogen; INR, international normalized ratio; PT, prothrombin time; APTT, activated partial thromboplastin time. | | | |

Supplementary Table 10. Multivariate Cox model adjusted with all covariates and IPTW for 28-day mortality of cohort

| **Characteristic** | **HR** | **95% CI** | ***p-*value** |
| --- | --- | --- | --- |
| Group |  |  |  |
| non-DEX | 1.00 | Reference |  |
| DEX | 0.57 | 0.33, 0.96 | 0.04 |
| Age | 1.04 | 1.03, 1.05 | <0.001 |
| Gender |  |  |  |
| Female | 1.00 | Reference |  |
| Male | 1.29 | 0.91, 1.83 | 0.15 |
| Race |  |  |  |
| White | 1.00 | Reference |  |
| other | 0.87 | 0.63, 1.20 | 0.4 |
| Cerebral laceration |  |  |  |
| YES | 1.00 | Reference |  |
| NO | 0.65 | 0.36, 1.17 | 0.15 |
| Subdural hematoma |  |  |  |
| YES | 1.00 | Reference |  |
| NO | 1.05 | 0.72, 1.53 | 0.8 |
| Diffuse brain injury |  |  |  |
| YES | 1.00 | Reference |  |
| NO | 0.66 | 0.35, 1.26 | 0.21 |
| APSIII | 1.03 | 1.01, 1.04 | <0.001 |
| SOFA score | 1.05 | 0.97, 1.13 | 0.21 |
| Charlson | 1.02 | 0.91, 1.14 | 0.7 |
| Mechanical ventilation use |  |  |  |
| YES | 1.00 | Reference |  |
| NO | 0.29 | 0.18, 0.46 | <0.001 |
| Vasopressor use |  |  |  |
| YES | 1.00 | Reference |  |
| NO | 1.03 | 0.68, 1.56 | 0.89 |
| Midazolam use |  |  |  |
| YES | 1.00 | Reference |  |
| NO | 2.35 | 1.23, 4.48 | 0.01 |
| Propofol use |  |  |  |
| YES | 1.00 | Reference |  |
| NO | 1.64 | 1.05, 2.56 | 0.03 |
| Fentanyl use |  |  |  |
| YES | 1.00 | Reference |  |
| NO | 1.14 | 0.77, 1.70 | 0.51 |
| HF |  |  |  |
| YES | 1.00 | Reference |  |
| NO | 0.95 | 0.54, 1.67 | 0.85 |
| Renal |  |  |  |
| YES | 1.00 | Reference |  |
| NO | 0.89 | 0.41, 1.93 | 0.76 |
| COPD |  |  |  |
| YES | 1.00 | Reference |  |
| NO | 1.50 | 0.86, 2.61 | 0.16 |
| Stroke |  |  |  |
| YES | 1.00 | Reference |  |
| NO | 0.72 | 0.44, 1.17 | 0.18 |
| Malignancy |  |  |  |
| YES | 1.00 | Reference |  |
| NO | 0.93 | 0.43, 2.01 | 0.85 |
| MAP | 1.01 | 1.00, 1.01 | 0.19 |
| Heart rate | 1.00 | 0.99, 1.01 | 0.64 |
| Temperature | 0.83 | 0.71, 0.97 | 0.02 |
| SPO2 | 1.01 | 0.97, 1.06 | 0.63 |
| GCS | 1.03 | 0.95, 1.12 | 0.43 |
| WBC | 1.01 | 1.00, 1.03 | 0.07 |
| Hemoglobin | 0.97 | 0.89, 1.06 | 0.53 |
| Platelet | 1.00 | 1.00, 1.00 | 0.15 |
| Glu | 1.00 | 1.00, 1.00 | 0.8 |
| Sodium | 1.00 | 0.96, 1.04 | 0.97 |
| Potassium | 1.02 | 0.83, 1.25 | 0.86 |
| Bicarbonate | 1.00 | 0.96, 1.04 | 0.89 |
| Chloride | 0.98 | 0.93, 1.02 | 0.28 |
| BUN | 1.01 | 1.00, 1.02 | 0.11 |
| Creatinine | 0.79 | 0.62, 1.00 | 0.05 |
| INR | 2.14 | 0.80, 5.75 | 0.13 |
| PT | 0.95 | 0.88, 1.02 | 0.13 |
| APTT | 1.01 | 1.00, 1.03 | 0.03 |
| Cerebral surgery |  |  |  |
| YES | 1.00 | Reference |  |
| NO | 0.63 | 0.41, 0.97 | 0.03 |
| Sepsis |  |  |  |
| YES | 1.00 | Reference |  |
| NO | 0.65 | 0.45, 0.93 | 0.02 |
| Vap |  |  |  |
| YES | 1.00 | Reference |  |
| NO | 1.77 | 1.08, 2.89 | 0.02 |
| Abbreviations: CI, Confidence Interval; HR, Hazard Ratio; DEX, dexmedetomidine; APS III, acute physiology score III; SOFA, sequential organ failure assessment; HF, heart failure; COPD, chronic obstructive pulmonary disease; MAP, mean arterial pressure; SPO2, oxygen saturation; GCS, Glasgow Coma Scale; VAP, ventilator-associated pneumonia; WBC, white blood cells; BUN, blood urea nitrogen; INR, international normalized ratio; PT, prothrombin time; APTT, activated partial thromboplastin time. | | | |

Supplementary Table 11. Multivariate Cox model adjusted with all covariates for hos mortality of original cohort

| **Characteristic** | **HR** | **95% CI** | ***p-*value** |
| --- | --- | --- | --- |
| Group |  |  |  |
| non-DEX | 1.00 | Reference |  |
| DEX | 0.25 | 0.15, 0.42 | <0.001 |
| Age | 1.04 | 1.03, 1.05 | <0.001 |
| Gender |  |  |  |
| Female | 1.00 | Reference |  |
| Male | 0.90 | 0.69, 1.17 | 0.43 |
| Race |  |  |  |
| White | 1.00 | Reference |  |
| other | 0.81 | 0.63, 1.04 | 0.09 |
| Cerebral laceration |  |  |  |
| YES | 1.00 | Reference |  |
| NO | 0.79 | 0.51, 1.23 | 0.3 |
| Subdural hematoma |  |  |  |
| YES | 1.00 | Reference |  |
| NO | 0.83 | 0.63, 1.10 | 0.2 |
| Diffuse brain injury |  |  |  |
| YES | 1.00 | Reference |  |
| NO | 0.61 | 0.36, 1.02 | 0.06 |
| APSIII | 1.02 | 1.01, 1.03 | <0.001 |
| SOFA score | 1.06 | 1.00, 1.13 | 0.06 |
| Charlson | 0.92 | 0.84, 1.02 | 0.1 |
| Mechanical ventilation use |  |  |  |
| YES | 1.00 | Reference |  |
| NO | 0.18 | 0.12, 0.27 | <0.001 |
| Vasopressor use |  |  |  |
| YES | 1.00 | Reference |  |
| NO | 0.63 | 0.45, 0.87 | 0.005 |
| Midazolam use |  |  |  |
| YES | 1.00 | Reference |  |
| NO | 2.50 | 1.53, 4.08 | <0.001 |
| Propofol use |  |  |  |
| YES | 1.00 | Reference |  |
| NO | 1.38 | 0.98, 1.94 | 0.06 |
| Fentanyl use |  |  |  |
| YES | 1.00 | Reference |  |
| NO | 1.36 | 0.98, 1.91 | 0.07 |
| HF |  |  |  |
| YES | 1.00 | Reference |  |
| NO | 1.43 | 0.97, 2.10 | 0.07 |
| Renal |  |  |  |
| YES | 1.00 | Reference |  |
| NO | 0.88 | 0.54, 1.44 | 0.62 |
| COPD |  |  |  |
| YES | 1.00 | Reference |  |
| NO | 0.82 | 0.50, 1.36 | 0.45 |
| Stroke |  |  |  |
| YES | 1.00 | Reference |  |
| NO | 0.76 | 0.52, 1.11 | 0.16 |
| Malignancy |  |  |  |
| YES | 1.00 | Reference |  |
| NO | 3.60 | 0.50, 26.1 | 0.21 |
| MAP | 1.00 | 1.00, 1.01 | 0.28 |
| Heart rate | 1.00 | 1.0, 1.01 | 0.66 |
| Temperature | 0.88 | 0.80, 0.97 | 0.01 |
| SPO2 | 0.98 | 0.95, 1.01 | 0.2 |
| GCS | 1.07 | 1.02, 1.12 | 0.01 |
| WBC | 1.02 | 1.01, 1.03 | <0.001 |
| Hemoglobin | 0.98 | 0.91, 1.04 | 0.49 |
| Platelet | 1.00 | 1.00, 1.00 | 0.86 |
| Glu | 1.00 | 1.00, 1.00 | 0.42 |
| Sodium | 1.06 | 1.02, 1.10 | 0.001 |
| Potassium | 1.33 | 1.13, 1.56 | <0.001 |
| Bicarbonate | 1.00 | 0.97, 1.04 | 0.81 |
| Chloride | 0.98 | 0.94, 1.01 | 0.15 |
| BUN | 1.01 | 1.00, 1.01 | 0.11 |
| Creatinine | 0.91 | 0.79, 1.06 | 0.25 |
| INR | 3.53 | 1.26, 9.92 | 0.02 |
| PT | 0.92 | 0.85, 1.00 | 0.06 |
| APTT | 1.01 | 0.99, 1.02 | 0.37 |
| Cerebral surgery |  |  |  |
| YES | 1.00 | Reference |  |
| NO | 0.56 | 0.30, 1.03 | 0.06 |
| Sepsis |  |  |  |
| YES | 1.00 | Reference |  |
| NO | 1.36 | 1.04, 1.78 | 0.02 |
| Vap |  |  |  |
| YES | 1.00 | Reference |  |
| NO | 3.84 | 2.33, 6.32 | <0.001 |
| Abbreviations: CI, Confidence Interval; HR, Hazard Ratio; DEX, dexmedetomidine; APS III, acute physiology score III; SOFA, sequential organ failure assessment; HF, heart failure; COPD, chronic obstructive pulmonary disease; MAP, mean arterial pressure; SPO2, oxygen saturation; GCS, Glasgow Coma Scale; VAP, ventilator-associated pneumonia; WBC, white blood cells; BUN, blood urea nitrogen; INR, international normalized ratio; PT, prothrombin time; APTT, activated partial thromboplastin time. | | | |

Supplementary Table 12. Multivariate Cox model adjusted with unbalanced covariates and IPTW for hos mortality of cohort

| **Characteristic** | **HR** | **95% CI** | ***p-*value** |
| --- | --- | --- | --- |
| Group |  |  |  |
| non-DEX | 1.00 | Reference |  |
| DEX | 0.28 | 0.17, 0.46 | <0.001 |
| Age | 1.03 | 1.02, 1.04 | <0.001 |
| Gender |  |  |  |
| Female | 1.00 | Reference |  |
| Male | 0.85 | 0.66, 1.09 | 0.19 |
| Race |  |  |  |
| White | 1.00 | Reference |  |
| other | 0.87 | 0.68, 1.11 | 0.26 |
| Subdural hematoma |  |  |  |
| YES | 1.00 | Reference |  |
| NO | 0.85 | 0.66, 1.10 | 0.22 |
| Diffuse brain injury |  |  |  |
| YES | 1.00 | Reference |  |
| NO | 0.67 | 0.40, 1.11 | 0.12 |
| SOFA score | 1.13 | 1.08, 1.19 | <0.001 |
| Charlson | 0.98 | 0.92, 1.06 | 0.68 |
| Mechanical ventilation use |  |  |  |
| YES | 1.00 | Reference |  |
| NO | 0.15 | 0.10, 0.22 | <0.001 |
| Vasopressor use |  |  |  |
| YES | 1.00 | Reference |  |
| NO | 0.57 | 0.41, 0.78 | <0.001 |
| Midazolam use |  |  |  |
| YES | 1.00 | Reference |  |
| NO | 2.25 | 1.40, 3.62 | <0.001 |
| Propofol use |  |  |  |
| YES | 1.00 | Reference |  |
| NO | 1.90 | 1.37, 2.63 | <0.001 |
| Fentanyl use |  |  |  |
| YES | 1.00 | Reference |  |
| NO | 1.25 | 0.91, 1.74 | 0.17 |
| HF |  |  |  |
| YES | 1.00 | Reference |  |
| NO | 1.47 | 1.00, 2.17 | 0.05 |
| MAP | 1.00 | 1.00, 1.01 | 0.56 |
| Temperature | 0.85 | 0.77, 0.94 | 0.001 |
| SPO2 | 0.98 | 0.95, 1.02 | 0.33 |
| Platelet | 1.00 | 1.00, 1.00 | 0.44 |
| Bicarbonate | 1.00 | 0.97, 1.03 | 0.9 |
| BUN | 1.01 | 1.00, 1.02 | <0.001 |
| INR | 4.10 | 1.38, 12.2 | 0.01 |
| PT | 0.91 | 0.83, 1.0 | 0.04 |
| APTT | 1.01 | 0.99, 1.02 | 0.31 |
| Cerebral surgery |  |  |  |
| YES | 1.00 | Reference |  |
| NO | 0.61 | 0.33, 1.11 | 0.1 |
| Sepsis |  |  |  |
| YES | 1.00 | Reference |  |
| NO | 1.37 | 1.05, 1.79 | 0.02 |
| Vap |  |  |  |
| YES | 1.00 | Reference |  |
| NO | 3.12 | 1.92, 5.06 | <0.001 |
| Abbreviations: CI, Confidence Interval; HR, Hazard Ratio; DEX, dexmedetomidine; APS III, acute physiology score III; SOFA, sequential organ failure assessment; HF, heart failure; COPD, chronic obstructive pulmonary disease; MAP, mean arterial pressure; SPO2, oxygen saturation; GCS, Glasgow Coma Scale; VAP, ventilator-associated pneumonia; WBC, white blood cells; BUN, blood urea nitrogen; INR, international normalized ratio; PT, prothrombin time; APTT, activated partial thromboplastin time. | | | |

Supplementary Table 13. Multivariate Cox model adjusted with covariates selected by Boruta algorithm for hos mortality of cohort

| **Characteristic** | **HR** | **95% CI** | ***p-*value** |
| --- | --- | --- | --- |
| Group |  |  |  |
| non-DEX | 1.00 | Reference |  |
| DEX | 0.25 | 0.15, 0.43 | <0.001 |
| Vap |  |  |  |
| YES | 1.00 | Reference |  |
| NO | 3.81 | 2.31, 6.26 | <0.001 |
| Sepsis |  |  |  |
| YES | 1.00 | Reference |  |
| NO | 1.30 | 1.00, 1.69 | 0.05 |
| APTT | 1.01 | 1.0, 1.02 | 0.26 |
| PT | 0.92 | 0.83, 1.01 | 0.06 |
| INR | 3.69 | 1.22, 11.2 | 0.02 |
| Creatinine | 0.92 | 0.79, 1.07 | 0.27 |
| BUN | 1.01 | 1.00, 1.01 | 0.11 |
| Chloride | 0.98 | 0.95, 1.01 | 0.24 |
| Bicarbonate | 1.00 | 0.97, 1.04 | 0.78 |
| Potassium | 1.32 | 1.13, 1.54 | <0.001 |
| Sodium | 1.05 | 1.01, 1.09 | 0.006 |
| Glu | 1.00 | 1.00, 1.00 | 0.27 |
| Platelet | 1.00 | 1.00, 1.00 | 0.9 |
| Hemoglobin | 0.98 | 0.92, 1.05 | 0.59 |
| WBC | 1.02 | 1.01, 1.03 | 0.001 |
| GCS | 1.06 | 1.01, 1.11 | 0.02 |
| SPO2 | 0.98 | 0.95, 1.01 | 0.21 |
| Temperature | 0.89 | 0.81, 0.98 | 0.02 |
| Heart rate | 1.00 | 1.00, 1.01 | 0.49 |
| Renal |  |  |  |
| YES | 1.00 | Reference |  |
| NO | 0.88 | 0.54, 1.43 | 0.61 |
| HF |  |  |  |
| YES | 1.00 | Reference |  |
| NO | 1.40 | 0.95, 2.05 | 0.09 |
| Fentanyl use |  |  |  |
| YES | 1.00 | Reference |  |
| NO | 1.34 | 0.97, 1.87 | 0.08 |
| Propofol use |  |  |  |
| YES | 1.00 | Reference |  |
| NO | 1.43 | 1.02, 2.00 | 0.04 |
| Midazolam use |  |  |  |
| YES | 1.00 | Reference |  |
| NO | 2.68 | 1.64, 4.38 | <0.001 |
| Vasopressor use |  |  |  |
| YES | 1.00 | Reference |  |
| NO | 0.64 | 0.46, 0.88 | 0.006 |
| Mechanical ventilation use |  |  |  |
| YES | 1.00 | Reference |  |
| NO | 0.15 | 0.10, 0.22 | <0.001 |
| Charlson | 0.94 | 0.86, 1.03 | 0.19 |
| SOFA score | 1.07 | 1.00, 1.13 | 0.05 |
| APSIII | 1.02 | 1.01, 1.03 | <0.001 |
| Subdural hematoma |  |  |  |
| YES | 1.00 | Reference |  |
| NO | 0.86 | 0.66, 1.13 | 0.28 |
| Age | 1.04 | 1.03, 1.05 | <0.001 |
| Abbreviations: CI, Confidence Interval; HR, Hazard Ratio; DEX, dexmedetomidine; APS III, acute physiology score III; SOFA, sequential organ failure assessment; HF, heart failure; COPD, chronic obstructive pulmonary disease; MAP, mean arterial pressure; SPO2, oxygen saturation; GCS, Glasgow Coma Scale; VAP, ventilator-associated pneumonia; WBC, white blood cells; BUN, blood urea nitrogen; INR, international normalized ratio; PT, prothrombin time; APTT, activated partial thromboplastin time. | | | |

Supplementary Table 14. Multivariate Cox model adjusted with all covariates and IPTW for hos mortality of cohort

| **Characteristic** | **HR** | **95% CI** | ***p-*value** |
| --- | --- | --- | --- |
| Group |  |  |  |
| non-DEX | 1.00 | Reference |  |
| DEX | 0.20 | 0.11, 0.37 | <0.001 |
| Age | 1.04 | 1.02, 1.05 | <0.001 |
| Gender |  |  |  |
| Female | 1.00 | Reference |  |
| Male | 0.87 | 0.63, 1.21 | 0.41 |
| Race |  |  |  |
| White | 1.00 | Reference |  |
| other | 0.70 | 0.50, 0.98 | 0.04 |
| Cerebral laceration |  |  |  |
| YES | 1.00 | Reference |  |
| NO | 0.55 | 0.31, 1.0 | 0.05 |
| Subdural hematoma |  |  |  |
| YES | 1.00 | Reference |  |
| NO | 1.01 | 0.71, 1.43 | 0.96 |
| Diffuse brain injury |  |  |  |
| YES | 1.00 | Reference |  |
| NO | 0.46 | 0.24, 0.87 | 0.02 |
| APSIII | 1.03 | 1.01, 1.04 | <0.001 |
| SOFA score | 1.06 | 0.99, 1.13 | 0.12 |
| Charlson | 0.93 | 0.81, 1.06 | 0.27 |
| Mechanical ventilation use |  |  |  |
| YES | 1.00 | Reference |  |
| NO | 0.27 | 0.15, 0.48 | <0.001 |
| Vasopressor use |  |  |  |
| YES | 1.00 | Reference |  |
| NO | 0.87 | 0.57, 1.33 | 0.51 |
| Midazolam use |  |  |  |
| YES | 1.00 | Reference |  |
| NO | 2.38 | 1.18, 4.80 | 0.02 |
| Propofol use |  |  |  |
| YES | 1.00 | Reference |  |
| NO | 1.22 | 0.77, 1.93 | 0.39 |
| Fentanyl use |  |  |  |
| YES | 1.00 | Reference |  |
| NO | 1.13 | 0.75, 1.70 | 0.57 |
| HF |  |  |  |
| YES | 1.00 | Reference |  |
| NO | 1.64 | 1.04, 2.58 | 0.03 |
| Renal |  |  |  |
| YES | 1.00 | Reference |  |
| NO | 0.95 | 0.54, 1.67 | 0.86 |
| COPD |  |  |  |
| YES | 1.00 | Reference |  |
| NO | 0.92 | 0.55, 1.52 | 0.74 |
| Stroke |  |  |  |
| YES | 1.00 | Reference |  |
| NO | 0.54 | 0.32, 0.92 | 0.02 |
| Malignancy |  |  |  |
| YES | 1.00 | Reference |  |
| NO | 11.4 | 0.61, 210 | 0.1 |
| MAP | 1.01 | 1.00, 1.01 | 0.09 |
| Heart rate | 1.00 | 0.99, 1.01 | 0.99 |
| Temperature | 0.89 | 0.76, 1.03 | 0.11 |
| SPO2 | 0.96 | 0.93, 1.00 | 0.05 |
| GCS | 1.07 | 1.01, 1.14 | 0.02 |
| WBC | 1.02 | 1.01, 1.04 | <0.001 |
| Hemoglobin | 1.02 | 0.94, 1.11 | 0.64 |
| Platelet | 1.00 | 1.00, 1.00 | 0.25 |
| Glu | 1.00 | 1.00, 1.00 | 0.93 |
| Sodium | 1.04 | 0.99, 1.08 | 0.1 |
| Potassium | 1.18 | 0.98, 1.42 | 0.09 |
| Bicarbonate | 1.02 | 0.98, 1.07 | 0.34 |
| Chloride | 0.96 | 0.91, 1.01 | 0.09 |
| BUN | 1.01 | 1.00, 1.02 | 0.07 |
| Creatinine | 0.88 | 0.74, 1.05 | 0.17 |
| INR | 2.80 | 1.15, 6.78 | 0.02 |
| PT | 0.93 | 0.88, 0.99 | 0.03 |
| APTT | 1.02 | 1.00, 1.03 | 0.03 |
| Cerebral surgery |  |  |  |
| YES | 1.00 | Reference |  |
| NO | 0.48 | 0.25, 0.90 | 0.02 |
| Sepsis |  |  |  |
| YES | 1.00 | Reference |  |
| NO | 1.18 | 0.85, 1.63 | 0.33 |
| Vap |  |  |  |
| YES | 1.00 | Reference |  |
| NO | 3.26 | 1.84, 5.75 | <0.001 |
| Abbreviations: CI, Confidence Interval; HR, Hazard Ratio; DEX, dexmedetomidine; APS III, acute physiology score III; SOFA, sequential organ failure assessment; HF, heart failure; COPD, chronic obstructive pulmonary disease; MAP, mean arterial pressure; SPO2, oxygen saturation; GCS, Glasgow Coma Scale; VAP, ventilator-associated pneumonia; WBC, white blood cells; BUN, blood urea nitrogen; INR, international normalized ratio; PT, prothrombin time; APTT, activated partial thromboplastin time. | | | |

Supplementary Table 15. Multivariate Cox model adjusted with all covariates for 1-year mortality of original cohort

| **Characteristic** | **HR** | **95% CI** | ***p-*value** |
| --- | --- | --- | --- |
| Group |  |  |  |
| non-DEX | 1.00 | Reference |  |
| DEX | 0.64 | 0.47, 0.87 | 0.005 |
| Age | 1.03 | 1.03, 1.04 | <0.001 |
| Gender |  |  |  |
| Female | 1.00 | Reference |  |
| Male | 0.99 | 0.83, 1.18 | 0.93 |
| Race |  |  |  |
| White | 1.00 | Reference |  |
| other | 0.90 | 0.76, 1.07 | 0.23 |
| Cerebral laceration |  |  |  |
| YES | 1.00 | Reference |  |
| NO | 0.76 | 0.55, 1.05 | 0.09 |
| Subdural hematoma |  |  |  |
| YES | 1.00 | Reference |  |
| NO | 0.90 | 0.75, 1.09 | 0.28 |
| Diffuse brain injury |  |  |  |
| YES | 1.00 | Reference |  |
| NO | 0.82 | 0.55, 1.24 | 0.35 |
| APSIII | 1.01 | 1.01, 1.02 | <0.001 |
| SOFA score | 1.07 | 1.02, 1.12 | 0.005 |
| Charlson | 1.06 | 1.01, 1.12 | 0.03 |
| Mechanical ventilation use |  |  |  |
| YES | 1.00 | Reference |  |
| NO | 0.27 | 0.20, 0.36 | <0.001 |
| Vasopressor use |  |  |  |
| YES | 1.00 | Reference |  |
| NO | 0.77 | 0.60, 0.99 | 0.04 |
| Midazolam use |  |  |  |
| YES | 1.00 | Reference |  |
| NO | 2.11 | 1.46, 3.06 | <0.001 |
| Propofol use |  |  |  |
| YES | 1.00 | Reference |  |
| NO | 1.64 | 1.25, 2.16 | <0.001 |
| Fentanyl use |  |  |  |
| YES | 1.00 | Reference |  |
| NO | 1.20 | 0.93, 1.55 | 0.15 |
| HF |  |  |  |
| YES | 1.00 | Reference |  |
| NO | 1.19 | 0.93, 1.53 | 0.16 |
| Renal |  |  |  |
| YES | 1.00 | Reference |  |
| NO | 1.06 | 0.79, 1.43 | 0.7 |
| COPD |  |  |  |
| YES | 1.00 | Reference |  |
| NO | 0.95 | 0.70, 1.29 | 0.72 |
| Stroke |  |  |  |
| YES | 1.00 | Reference |  |
| NO | 0.74 | 0.57, 0.98 | 0.03 |
| Malignancy |  |  |  |
| YES | 1.00 | Reference |  |
| NO | 1.29 | 0.53, 3.13 | 0.58 |
| MAP | 1.01 | 1.00, 1.01 | 0.02 |
| Heart rate | 1.00 | 1.00, 1.01 | 0.09 |
| Temperature | 0.78 | 0.72, 0.84 | <0.001 |
| SPO2 | 1.01 | 0.99, 1.04 | 0.36 |
| GCS | 1.00 | 0.96, 1.03 | 0.82 |
| WBC | 1.01 | 1.00, 1.02 | 0.01 |
| Hemoglobin | 0.93 | 0.89, 0.98 | 0.003 |
| Platelet | 1.00 | 1.00, 1.00 | 0.26 |
| Glu | 1.00 | 1.00, 1.00 | 0.24 |
| Sodium | 1.05 | 1.03, 1.08 | <0.001 |
| Potassium | 1.15 | 1.02, 1.29 | 0.02 |
| Bicarbonate | 1.00 | 0.97, 1.02 | 0.75 |
| Chloride | 0.96 | 0.94, 0.99 | 0.003 |
| BUN | 1.00 | 1.00, 1.01 | 0.31 |
| Creatinine | 0.94 | 0.85, 1.04 | 0.23 |
| INR | 2.33 | 0.99, 5.47 | 0.05 |
| PT | 0.94 | 0.87, 1.01 | 0.09 |
| APTT | 1.01 | 1.00, 1.02 | 0.21 |
| Cerebral surgery |  |  |  |
| YES | 1.00 | Reference |  |
| NO | 0.94 | 0.73, 1.22 | 0.65 |
| Sepsis |  |  |  |
| YES | 1.00 | Reference |  |
| NO | 0.93 | 0.77, 1.11 | 0.42 |
| Vap |  |  |  |
| YES | 1.00 | Reference |  |
| NO | 1.15 | 0.85, 1.57 | 0.37 |
| Abbreviations: CI, Confidence Interval; HR, Hazard Ratio; DEX, dexmedetomidine; APS III, acute physiology score III; SOFA, sequential organ failure assessment; HF, heart failure; COPD, chronic obstructive pulmonary disease; MAP, mean arterial pressure; SPO2, oxygen saturation; GCS, Glasgow Coma Scale; VAP, ventilator-associated pneumonia; WBC, white blood cells; BUN, blood urea nitrogen; INR, international normalized ratio; PT, prothrombin time; APTT, activated partial thromboplastin time. | | | |

Supplementary Table 16. Multivariate Cox model adjusted with unbalanced covariates and IPTW for 1-year mortality of cohort

| **Characteristic** | **HR** | **95% CI** | ***p-*value** |
| --- | --- | --- | --- |
| Group |  |  |  |
| non-DEX | 1.00 | Reference |  |
| DEX | 0.67 | 0.50, 0.92 | 0.01 |
| Age | 1.03 | 1.02, 1.04 | <0.001 |
| Gender |  |  |  |
| Female | 1.00 | Reference |  |
| Male | 0.88 | 0.74, 1.04 | 0.14 |
| Race |  |  |  |
| White | 1.00 | Reference |  |
| other | 0.94 | 0.79, 1.11 | 0.45 |
| Subdural hematoma |  |  |  |
| YES | 1.00 | Reference |  |
| NO | 0.94 | 0.79, 1.13 | 0.52 |
| Diffuse brain injury |  |  |  |
| YES | 1.00 | Reference |  |
| NO | 0.89 | 0.59, 1.33 | 0.57 |
| SOFA score | 1.15 | 1.11, 1.19 | <0.001 |
| Charlson | 1.10 | 1.05, 1.15 | <0.001 |
| Mechanical ventilation use |  |  |  |
| YES | 1.00 | Reference |  |
| NO | 0.24 | 0.18, 0.31 | <0.001 |
| Vasopressor use |  |  |  |
| YES | 1.00 | Reference |  |
| NO | 0.77 | 0.60, 0.98 | 0.04 |
| Midazolam use |  |  |  |
| YES | 1.00 | Reference |  |
| NO | 2.10 | 1.46, 3.02 | <0.001 |
| Propofol use |  |  |  |
| YES | 1.00 | Reference |  |
| NO | 2.03 | 1.56, 2.63 | <0.001 |
| Fentanyl use |  |  |  |
| YES | 1.00 | Reference |  |
| NO | 1.16 | 0.91, 1.48 | 0.23 |
| HF |  |  |  |
| YES | 1.00 | Reference |  |
| NO | 1.19 | 0.93, 1.52 | 0.16 |
| MAP | 1.00 | 1.00, 1.01 | 0.05 |
| Temperature | 0.76 | 0.70, 0.82 | <0.001 |
| SPO2 | 1.01 | 0.99, 1.04 | 0.34 |
| Platelet | 1.00 | 1.00, 1.00 | 0.58 |
| Bicarbonate | 1.01 | 0.99, 1.03 | 0.49 |
| BUN | 1.01 | 1.00, 1.01 | 0.03 |
| INR | 2.82 | 1.07, 7.41 | 0.04 |
| PT | 0.93 | 0.85, 1.01 | 0.08 |
| APTT | 1.00 | 0.99, 1.01 | 0.6 |
| Cerebral surgery |  |  |  |
| YES | 1.00 | Reference |  |
| NO | 0.98 | 0.76, 1.27 | 0.9 |
| Sepsis |  |  |  |
| YES | 1.00 | Reference |  |
| NO | 0.88 | 0.74, 1.06 | 0.19 |
| Vap |  |  |  |
| YES | 1.00 | Reference |  |
| NO | 1.06 | 0.78, 1.43 | 0.71 |
| Abbreviations: CI, Confidence Interval; HR, Hazard Ratio; DEX, dexmedetomidine; APS III, acute physiology score III; SOFA, sequential organ failure assessment; HF, heart failure; COPD, chronic obstructive pulmonary disease; MAP, mean arterial pressure; SPO2, oxygen saturation; GCS, Glasgow Coma Scale; VAP, ventilator-associated pneumonia; WBC, white blood cells; BUN, blood urea nitrogen; INR, international normalized ratio; PT, prothrombin time; APTT, activated partial thromboplastin time. | | | |

Supplementary Table 17. Multivariate Cox model adjusted with covariates selected by Boruta algorithm for 1-year mortality of cohort

| **Characteristic** | **HR** | **95% CI** | ***p-*value** |
| --- | --- | --- | --- |
| Group |  |  |  |
| non-DEX | 1.00 | Reference |  |
| DEX | 0.64 | 0.47, 0.88 | 0.005 |
| Vap |  |  |  |
| YES | 1.00 | Reference |  |
| NO | 1.12 | 0.82, 1.52 | 0.47 |
| Sepsis |  |  |  |
| YES | 1.00 | Reference |  |
| NO | 0.91 | 0.76, 1.10 | 0.34 |
| APTT | 1.01 | 1.00, 1.02 | 0.18 |
| PT | 0.93 | 0.86, 1.01 | 0.07 |
| INR | 2.61 | 1.05, 6.47 | 0.04 |
| Creatinine | 0.95 | 0.86, 1.05 | 0.32 |
| BUN | 1.00 | 1.00, 1.01 | 0.28 |
| Chloride | 0.97 | 0.94, 0.99 | 0.007 |
| Bicarbonate | 1.00 | 0.97, 1.02 | 0.89 |
| Potassium | 1.14 | 1.01, 1.28 | 0.03 |
| Sodium | 1.05 | 1.02, 1.08 | <0.001 |
| Glu | 1.00 | 1.00, 1.00 | 0.14 |
| Platelet | 1.00 | 1.00, 1.00 | 0.37 |
| Hemoglobin | 0.94 | 0.90, 0.98 | 0.006 |
| WBC | 1.01 | 1.00, 1.02 | 0.01 |
| GCS | 0.99 | 0.95, 1.02 | 0.45 |
| SPO2 | 1.01 | 0.99, 1.04 | 0.36 |
| Temperature | 0.78 | 0.72, 0.84 | <0.001 |
| Heart rate | 1.00 | 1.00, 1.01 | 0.03 |
| Renal |  |  |  |
| YES | 1.00 | Reference |  |
| NO | 1.05 | 0.78, 1.41 | 0.73 |
| HF |  |  |  |
| YES | 1.00 | Reference |  |
| NO | 1.19 | 0.93, 1.52 | 0.16 |
| Fentanyl use |  |  |  |
| YES | 1.00 | Reference |  |
| NO | 1.16 | 0.90, 1.49 | 0.24 |
| Propofol use |  |  |  |
| YES | 1.00 | Reference |  |
| NO | 1.66 | 1.27, 2.18 | <0.001 |
| Midazolam use |  |  |  |
| YES | 1.00 | Reference |  |
| NO | 2.19 | 1.51, 3.16 | <0.001 |
| Vasopressor use |  |  |  |
| YES | 1.00 | Reference |  |
| NO | 0.79 | 0.62, 1.02 | 0.07 |
| Mechanical ventilation use |  |  |  |
| YES | 1.00 | Reference |  |
| NO | 0.26 | 0.20, 0.34 | <0.001 |
| Charlson | 1.08 | 1.02, 1.14 | 0.006 |
| SOFA score | 1.07 | 1.02, 1.12 | 0.006 |
| APSIII | 1.01 | 1.00, 1.02 | 0.002 |
| Subdural hematoma |  |  |  |
| YES | 1.00 | Reference |  |
| NO | 0.95 | 0.80, 1.13 | 0.58 |
| Age | 1.03 | 1.03, 1.04 | <0.001 |
| Abbreviations: CI, Confidence Interval; HR, Hazard Ratio; DEX, dexmedetomidine; APS III, acute physiology score III; SOFA, sequential organ failure assessment; HF, heart failure; COPD, chronic obstructive pulmonary disease; MAP, mean arterial pressure; SPO2, oxygen saturation; GCS, Glasgow Coma Scale; VAP, ventilator-associated pneumonia; WBC, white blood cells; BUN, blood urea nitrogen; INR, international normalized ratio; PT, prothrombin time; APTT, activated partial thromboplastin time. | | | |

Supplementary Table 18. Multivariate Cox model adjusted with all covariates and IPTW for 1-year mortality of cohort

| **Characteristic** | **HR** | **95% CI** | ***p-*value** |
| --- | --- | --- | --- |
| Group |  |  |  |
| non-DEX | 1.00 | Reference |  |
| DEX | 0.72 | 0.50, 1.04 | 0.08 |
| Age | 1.05 | 1.03, 1.06 | <0.001 |
| Gender |  |  |  |
| Female | 1.00 | Reference |  |
| Male | 1.37 | 1.06, 1.76 | 0.02 |
| Race |  |  |  |
| White | 1.00 | Reference |  |
| other | 0.80 | 0.61, 1.04 | 0.1 |
| Cerebral laceration |  |  |  |
| YES | 1.00 | Reference |  |
| NO | 0.79 | 0.44, 1.43 | 0.44 |
| Subdural hematoma |  |  |  |
| YES | 1.00 | Reference |  |
| NO | 1.23 | 0.95, 1.61 | 0.12 |
| Diffuse brain injury |  |  |  |
| YES | 1.00 | Reference |  |
| NO | 0.73 | 0.43, 1.22 | 0.23 |
| APSIII | 1.02 | 1.01, 1.03 | 0.004 |
| SOFA score | 1.04 | 0.97, 1.11 | 0.27 |
| Charlson | 1.09 | 1.01, 1.18 | 0.03 |
| Mechanical ventilation use |  |  |  |
| YES | 1.00 | Reference |  |
| NO | 0.41 | 0.27, 0.64 | <0.001 |
| Vasopressor use |  |  |  |
| YES | 1.00 | Reference |  |
| NO | 1.08 | 0.73, 1.58 | 0.71 |
| Midazolam use |  |  |  |
| YES | 1.00 | Reference |  |
| NO | 1.79 | 1.05, 3.07 | 0.03 |
| Propofol use |  |  |  |
| YES | 1.00 | Reference |  |
| NO | 1.79 | 1.17, 2.75 | 0.007 |
| Fentanyl use |  |  |  |
| YES | 1.00 | Reference |  |
| NO | 0.95 | 0.68, 1.31 | 0.74 |
| HF |  |  |  |
| YES | 1.00 | Reference |  |
| NO | 1.25 | 0.89, 1.77 | 0.19 |
| Renal |  |  |  |
| YES | 1.00 | Reference |  |
| NO | 0.94 | 0.58, 1.50 | 0.78 |
| COPD |  |  |  |
| YES | 1.00 | Reference |  |
| NO | 1.10 | 0.74, 1.63 | 0.65 |
| Stroke |  |  |  |
| YES | 1.00 | Reference |  |
| NO | 0.89 | 0.56, 1.43 | 0.63 |
| Malignancy |  |  |  |
| YES | 1.00 | Reference |  |
| NO | 0.71 | 0.40, 1.25 | 0.23 |
| MAP | 1.01 | 1.00, 1.01 | 0.07 |
| Heart rate | 1.00 | 0.99, 1.01 | 0.96 |
| Temperature | 0.75 | 0.65, 0.87 | <0.001 |
| SPO2 | 1.01 | 0.98, 1.04 | 0.44 |
| GCS | 0.97 | 0.92, 1.03 | 0.35 |
| WBC | 1.00 | 0.99, 1.02 | 0.59 |
| Hemoglobin | 0.92 | 0.85, 0.99 | 0.02 |
| Platelet | 1.00 | 1.00, 1.00 | 0.91 |
| Glu | 1.00 | 1.00, 1.00 | 0.71 |
| Sodium | 1.01 | 0.97, 1.05 | 0.6 |
| Potassium | 1.00 | 0.83, 1.20 | 0.99 |
| Bicarbonate | 1.00 | 0.96, 1.03 | 0.87 |
| Chloride | 0.96 | 0.92, 1.00 | 0.03 |
| BUN | 1.01 | 1.00, 1.02 | 0.08 |
| Creatinine | 0.86 | 0.74, 1.00 | 0.04 |
| INR | 1.67 | 0.64, 4.33 | 0.29 |
| PT | 0.96 | 0.90, 1.02 | 0.21 |
| APTT | 1.01 | 1.00, 1.03 | 0.01 |
| Cerebral surgery |  |  |  |
| YES | 1.00 | Reference |  |
| NO | 0.64 | 0.46, 0.90 | 0.01 |
| Sepsis |  |  |  |
| YES | 1.00 | Reference |  |
| NO | 0.74 | 0.55, 1.01 | 0.06 |
| Vap |  |  |  |
| YES | 1.00 | Reference |  |
| NO | 0.89 | 0.63, 1.26 | 0.51 |
| Abbreviations: CI, Confidence Interval; HR, Hazard Ratio; DEX, dexmedetomidine; APS III, acute physiology score III; SOFA, sequential organ failure assessment; HF, heart failure; COPD, chronic obstructive pulmonary disease; MAP, mean arterial pressure; SPO2, oxygen saturation; GCS, Glasgow Coma Scale; VAP, ventilator-associated pneumonia; WBC, white blood cells; BUN, blood urea nitrogen; INR, international normalized ratio; PT, prothrombin time; APTT, activated partial thromboplastin time. | | | |
